# Dietary Flavonoids in the Prevention of T2D: An Overview

**DOI:** 10.3390/nu10040438

**Published:** 2018-03-31

**Authors:** Hana Alkhalidy, Yao Wang, Dongmin Liu

**Affiliations:** 1 Department of Human Nutrition, Foods and Exercise, College of Agricultural and Life Sciences, Virginia Tech, Blacksburg, VA 24060, USA; hkhaldi@vt.edu (H.A.); yaow@vt.edu (Y.W.); 2 Department of Nutrition and Food Technology, Faculty of Agriculture, Jordan University of Science and Technology, Irbid 22110, Jordan

**Keywords:** flavonoids, type 2 diabetes, insulin resistance

## Abstract

Type 2 diabetes (T2D) is a progressive metabolic disease that is increasing in prevalence globally. It is well established that insulin resistance (IR) and a progressive decline in functional β-cell mass are hallmarks of developing T2D. Obesity is a leading pathogenic factor for developing IR. Constant IR will progress to T2D when β-cells are unable to secret adequate amounts of insulin to compensate for decreased insulin sensitivity. Recently, a considerable amount of research has been devoted to identifying naturally occurring anti-diabetic compounds that are abundant in certain types of foods. Flavonoids are a group of polyphenols that have drawn great interest for their various health benefits. Results from many clinical and animal studies demonstrate that dietary intake of flavonoids might be helpful in preventing T2D, although cellular and molecular mechanisms underlying these effects are still not completely understood. This review discusses our current understanding of the pathophysiology of T2D and highlights the potential anti-diabetic effects of flavonoids and mechanisms of their actions.

## 1. Introduction

The prevalence of diabetes is rapidly rising. In 2012, 9.3% of the US population was diabetic [1], and this number is expected to double by 2050 [2]. In addition, the cost of treating diabetes and its complications is an increasing economic burden [3]. In the U.S, the estimated annual cost of diagnosed diabetes increased from $174 billion to $245 billion between the years of 2007 and 2012 [4]. Type 2 diabetes (T2D) is a progressive metabolic disorder with a characteristic hyperglycemia accompanied by abnormalities in carbohydrate [5], lipid [6], and protein metabolism [7]. The cascade of events that lead to the development of T2D has long been the subject of debate [8,9]. However, it is well recognized that insulin resistance (IR), defects in insulin action, and impaired β-cell function are key features in T2D [10]. Subjects with IR will progress to overt diabetes if β-cells fail to secrete adequate amounts of insulin to compensate for the defects in its action [11]. Thus, β-cell failure plays a central role in the development T2D. 

There is a strong evidence suggesting that hyperglycemia plays a major role in the pathogenesis of diabetic complications that affects various organs in the body [12,13]. Hyperglycemia increases glucose metabolism, which can lead to excessive reactive oxygen species (ROS) production that will impair cell function and survival [14]. Moreover, hyperglycemia, in turn, aggravates IR, thereby forming a vicious circle [15]. Improved glucose control was shown to reduce the risk of diabetic complications such as microvascular complications [16,17,18]. The type and starting time of treatment upon diagnosis was associated with the risk of developing the complications [17,19]. In addition to insulin therapy, diabetes treatments include inhibiting oligo- and disaccharide degradation, reducing insulin demand, stimulating endogenous insulin secretion, and enhancing insulin action at target tissues [20,21].

There is a considerable amount of knowledge about the means to prevent and treat T2D, however, this knowledge is not fully applied or practiced in public health [22]. Also, some diabetes therapies have side effects [21] which necessitates the search for naturally occurring, cheaper, and safer compounds for preventing T2D. Flavonoids, polyphenolic compounds abundant in some fruits, vegetables, and medicinal herbs, exert many beneficial effects in various chronic diseases including diabetes [23]. This review will first summarize the current knowledge of IR and pathogenesis of pancreatic β-cell dysfunction in the context of the development of T2D. We will then briefly discuss the bioactivity of the various classes of flavonoids, including the pathways of absorption and metabolism. Primarily, this review will compile recent information from experimental studies, epidemiological observations, and clinical trials on the effects of flavonoids on T2D and the mechanisms of their actions.

## 2. Type 2 Diabetes

Glucose homeostasis is tightly controlled by the harmonization of multiple pathways in the postprandial (fed) and post-absorptive (fasted) states [24]. After the ingestion of a meal, the majority of glucose is transported into enterocytes lining the wall of the small intestine and then from the enterocytes into the portal vein by the glucose transporters sodium-dependent glucose cotransporter 1 (SGLT1) (brush-border membrane facing the lumen) and glucose transporter 2 (GLUT2) (basolateral membrane facing the bloodstream), respectively [25]. The rise in glucose in the portal vein (drains directly into liver before entering general circulation) induces glucose uptake facilitated by GLUT2, followed by phosphorylation of glucose to glucose-6-phosphate by glucokinase (GK) in the liver [26], hence increasing glucose clearance and storage by the liver before it reaches the circulation [27]. The activation of GK is accompanied by inhibition of a key gluconeogenic enzyme, glucose-6-phosphatase (G6Pase), which is responsible for the last step in gluconeogenesis and glycogenolysis by dephosphorylating glucose to its free form [28]. Consequently, the rate of hepatic glucose production is suppressed [29]. Meanwhile, pancreatic β-cells sense increased circulating glucose and subsequently elevate glucose influx into the cells via GLUT2, which is the only glucose transporter expressed in β-cells. GLUT2 has a low substrate affinity, and mobilization of GLUT2 to the plasma membrane is insulin-independent, which may be necessary for ensuring efficient and high glucose uptake into the cells [30]. It is well characterized that glucose induces insulin secretion through glycolysis and mitochondrial oxidation in the cells, which increase intracellular adenosine triphosphate/adenosine diphosphate (ATP/ADP) ratio, sequentially leading to closure of K_ATP_ channels, depolarization of voltage-gated l-type Ca^2+^ channels on the plasma membrane, Ca^2+^ influx, and ultimate activation of exocytosis of insulin-containing granules [31,32,33]. 

After insulin travels from pancreatic β-cells into the portal vein, it then rapidly acts on tissues like the liver to increase glycogen synthesis and reduce glycogenolysis while simultaneously inhibiting gluconeogenesis [34,35]. Skeletal muscle tissue makes a major contribution to insulin-dependent glucose uptake, accounting for about 30% of total glucose clearance as compared with about 39% by splanchnic tissues, primarily by the liver [36]. Glucose uptake is mediated by the combined influence of glucose concentrations (concentration-dependent facilitated diffusion via transporters) and insulin signaling (leading to increased membrane localization of transporters like glucose transporter 4, GLUT4) [37]. Intracellular glucose is phosphorylated by hexokinase in the muscle myofibers [38] and is then routed to different destinations such as glycolysis, oxidation, or glycogen synthesis [39,40]. In the fasting state however, glycogen in the liver is hydrolyzed to release glucose, and gluconeogenesis is increased, in order to maintain glucose homeostasis [41]. When blood glucose levels are low, the secretion of glucagon from pancreatic α-cells increases, which leads to increased glucose production and thus glucose output into the blood [42]. Glucagon acts to enhance the rate of glycogen breakdown and gluconeogenesis in the liver through modulating the transcription and activity of key glucogenic enzymes such as G6Pase and phosphoenolpyruvate carboxykinase (PEPCK) [43]. The liver is the primary supplier of glucose during fasting and is responsible for about 90% of the overall produced glucose, while the kidneys produce the remaining percentage [44]. Although the muscle stores glycogen [45], it cannot be released to the circulation as free glucose due to the lack of G6Pase [46]. However, peripheral tissues including skeletal muscle and adipose tissues can supply the liver with glucogenic precursors such as amino acids and glycerol, pathways that are blocked in the presence of insulin [47].

Insulin acts on target tissues such as muscle, liver, and adipose tissues [9]. It binds to its plasma membrane receptor, a tyrosine kinase receptor, which then triggers a chain of events that eventually affects many processes in the short and long term including glucose and lipid metabolic homeostasis [48]. However, IR in peripheral tissues such as skeletal muscle reduces glucose uptake, utilization, and storage [49]. Moreover, IR in the liver can result in excessive hepatic glucose production because of increased gluconeogenesis and glycogenolysis, which make a significant contribution to fasting and postprandial hyperglycemia, the hallmarks of T2D [50,51,52]. Likewise, IR may impair the function of the kidneys [53], thereby contributing to the development of hyperglycemia in T2D, as kidneys also play a role in regulating glucose homeostasis [54]. In normal subjects, kidneys act similarly to the liver in maintaining glucose homeostasis through glucose uptake and production in the fed [55] and fasting state [56], respectively. There is a reciprocal relationship between liver and kidneys to maintain glucose homeostasis referred to as hepatorenal glucose reciprocity [57,58]. In animal models of T2D, glucose transporters in kidneys are upregulated [59], which results in increased glucose reabsorption [60]. Similar findings were reported in T2D patients suggesting that the increased activity of glucose transporters in kidneys might make substantial contribution to hyperglycemia in T2D [61].

### 2.1. IR and T2D

The underlying mechanisms that lead to the development of IR are still an active area of investigation. Many genetic and environmental factors are involved in the development of IR [62]. One of the biggest difficulties in investigating IR is the accompanying metabolic abnormalities referred to as IR syndrome (IRS), or more commonly metabolic syndrome (MetS) [63], which also increases the risk of developing T2D [64]. Elevated plasma free fatty acids (FFAs) associated with obesity [65] or independent of obesity, i.e., consumption of a large amount of dietary fat, may impair the insulin signaling pathway leading to IR in the muscle and liver [66,67]. Other factors that play a role in inducing IR are genetic polymorphisms, abnormal cytokine and adipokine production, and endothelial dysfunction [65], which will be discussed in more detail in this review.

#### 2.1.1. IR in Muscle and Development of T2D

There is strong evidence suggesting that IR in skeletal muscle is a primary risk factor for T2D [68,69]. For glucose uptake, storage and utilization in the muscle, insulin activation of the insulin receptor substrate (IRS)-1/phosphatidylinositol (PI)-3 kinase (PI3K)/kinase B (or Akt) pathway is required [70]. However, this pathway was reported to be impaired in subjects that were genetically predisposed to developing T2D [71], and in T2D patients [72]. Due to this defect in signaling, IR develops in the muscle leading to a decrease in glucose uptake and utilization (mainly in glycogen synthesis) [73].

Obesity is a major risk factor for developing T2D, particularly once associated with IR [74]. In obesity, plasma FFAs are chronically elevated [65], which in muscle, can directly inhibit insulin activation of the IRS-1/PI3K/Akt pathway leading to reduced glucose uptake and phosphorylation, and decreased glycogen synthase activity [66,67]. One of the pathways connected to this alteration in insulin signaling is the diacylglycerol (DAG)/protein kinase C (PKC) pathway. Elevation of FFAs was associated with an increase in DAG, which in turn activates PKC-θ, -β2 and -δ [75]. The activation of these isoforms phosphorylates IRS1 on Ser^307^, which interfers with insulin-stimulated phosphorylation of IRS1, thus inhibiting insulin signaling [76]. The excess deposition of intramyocellular lipid (IMCL), which is associated with IR in lean and obese subjects [77], may play a role in inducing IR by activating the DAG/PKC pathway similar to FFAs. However, IMCL activates another PKC isoform, PKC-ε, which induces IR [78,79]. Increased activity of these PKC isoforms in muscle was observed in animal models of obesity and T2D [80,81].

Mitochondria generate energy via oxidative phosphorylation of nutrients such as glucose and fatty acids and thus play an important role in the regulation of cellular metabolism [82]. Mitochondrial dysfunction has been implicated in the development of IR. Indeed, defects in glucose uptake in the muscle of T2D subjects was associated with decreased glucose oxidation [83] and impaired fatty acid metabolism [84], indicative of mitochondrial dysfunction. The decrease in glucose uptake and utilization observed in the muscles of IR subjects may be the cause or the result of the mitochondrial dysfunction. For instance, impaired mitochondrial activity in muscles of IR subjects may lead to increased IMCL deposition which subsequently results in the development of IR [85]. Moreover, downregulation of genes encoding key enzymes for mitochondrial oxidative phosphorylation in the muscle is also linked to IR and T2D [86]. However, the cause and effect relationship between IR and mitochondrial function is still elusive. For example, IR increases ROS production leading to impaired mitochondrial function [87]. For more information about the role of dysfunctional mitochondria in developing IR and T2D, please refer to the comprehensive review by Szendroedi et al. [88].

The endothelium is essential for regulating vascular tone, and endothelial dysfunction impairs the release of nitric oxide (NO), thus affecting vascular homeostasis [89]. In particular, dysfunction of the peripheral vascular endothelium plays a role in the pathogenesis of IR [90]. This vascular dysfunction results in reduced expansion of the capillary network in the major target tissues of insulin such as the skeletal muscle, thereby reducing blood flow and supply of insulin to these tissues, which then subsequently impairs glucose and lipid metabolism [91].

#### 2.1.2. IR in Liver and Development of T2D

The liver has a vital role in maintaining glucose homeostasis in both the fed and fasting states with a major contribution to the latter [24]. Increased hepatic glucose production is considered to be one of the early pathological changes leading to T2D in humans [50,92,93]. There is evidence suggesting that such hepatic metabolic abnormalities in T2D are caused primarily by IR [94]. Insulin is involved in the direct and indirect suppression of hepatic glucose production, which is impaired when there is hepatic IR. In healthy subjects, insulin can suppress the flux of the glucogenic precursors from peripheral tissues such as nonesterified fatty acids to the liver, by promoting lipogenesis and inhibiting lipolysis in adipose tissue [95]. Also, insulin inhibits glucagon production and subsequently reduces the expression and activity of glucogenic enzymes such as PEPCK and G6Pase [96]. However, in IR these indirect pathways are not blocked, in part because insulin is unable to adequately regulate the gene expression and function of PEPCK [97] and G6Pase [98], leading to excessive hepatic glucose production through gluconeogenesis and glycogenolysis, thereby contributing to fasting hyperglycemia [50,51,52]. Increased hepatic gluconeogenesis, in particular, is considered one of the early pathological changes in newly diagnosed T2D subjects [50]. The activation of Akt by insulin contributes to the control of hepatic glucose metabolism [99], reduction in hepatic glucose output by stimulating glycogen synthesis [100,101], and downregulation of PEPCK and G6Pase gene expression [102]. In one report, gene expression of PEPCK and G6Pase was not changed nor was associated with fasting hyperglycemia in T2D subjects [103]. However, morbidly obese patients with T2D had an increase in hepatic glucose production which was associated with IR and an increase in liver G6Pase activity [104], suggesting that the increased gluconeogenic enzymes’ activities rather than their expression may play a larger role for gluconeogenesis in IR and diabetic subjects.

The forkhead box O (FoxO) family of transcription factors play important roles in a variety of physiological and pathological processes. Altered FoxO expression was associated with several metabolic diseases including diabetes. FoxO1 plays a role in mediating hormone-induced hepatic gluconeogenesis [105], by activating transcription of genes encoding PEPCK and G6Pase [106]. Liver-specific FoxO1 knockout mice displayed fasting hypoglycemia associated with reduced expression of gluconeogenic genes [107]. In addition, FoxO3 and FoxO4 enhance FoxO1-induced hepatic glucose production in mice [107], suggesting that these FoxO transcription factors collectively contribute to the control of hepatic glucose production. The action of FoxO1 in the liver is suppressed by insulin-mediated pathways but is augmented by peroxisome proliferative activated receptor-gamma co-activator-1alpha (PGC-1α), which serves as a transcriptional co-activator for FoxO1, thereby augmenting the expression of gluconeogenic genes. [108]. Interestingly, PGC-1α expression was upregulated in the liver of T2D subjects [109], which might partially explain the link between PGC-1α, FoxO1, and glucose production in diabetes. Recently, another FoxO family member, FoxO6 was found to play a similar role as FoxO1 in regulating gluconeogenesis, as increased FoxO6 activity in the liver promoted gluconeogenesis and increased fasting blood glucose levels, whereas FoxO6 deletion in the liver reduced gluconeogenesis, resulting in fasting hypoglycemia. However, unlike FoxO1, the transcriptional activity of FoxO6 is largely inhibited by insulin through activation of the Akt-mediated pathway, which leads to its nuclear exclusion and degradation in hepatocytes [110].

IR in skeletal muscle can cause the development of IR in the liver. In muscle tissues with IR, the failure of insulin to activate glycogen synthesis leads to a repartitioning of energy substrates to de novo lipogenesis in the liver [111], which lead to the accumulation of fat in hepatocytes [112]. Moreover, excess intrahepatocellular lipid (IHCL) accumulation and elevated FFAs may cause a wide spectrum of dysfunctionalities in the liver, including IR. On the other hand, hepatic IR may precede the development of IR in the muscle. For instance, high fat (HF) overfeeding induced hepatic IR in healthy subjects, along with elevated fasting blood glucose levels and insulin secretion before the development of IR in the muscle [113]. Similarly, short-term HF diet feeding in rats resulted in hepatic IR and hepatic steatosis, which was independent of IR in skeletal muscle. However, it was associated with activation of PKC-ε, attenuated insulin-stimulated signaling pathways, increased gluconeogenesis, and decreased insulin-dependent activation of glycogen synthase [114]. The activation of PKC-ε by FFAs and IHCL in the liver was consistent with the activation of this isoform in the muscle. In this regard, DAG may play a role in the activation of PKC which then inhibits insulin activation of IRS and subsequently its initiated signaling, thereby leading to the development of hepatic IR and hyperglycemia [111,115]. The accumulation of lipids in the liver and the promotion of fatty acid oxidation may increase ROS production, which then impairs mitochondrial functions and induce abnormalities in liver function [116].

#### 2.1.3. Relationship between Obesity, Inflammation, IR and Development of T2D

Obesity, a condition of fat accumulation in the body defined as having a body mass index (BMI) ≥30, is a worldwide epidemic that is still increasing globally [117]. Obesity is strongly associated with the development of IR, dyslipidemia, and T2D [118]. It is well recognized that obesity is partly a chronic inflammatory disease. Indeed, studies have suggested a direct connection between obesity and systematic inflammation due to the upregulation of key genes associated with inflammation [119], and the increased secretion of inflammatory markers from white adipose tissue (WAT) [120]. Abdominal obesity, in particular, is associated with many chronic diseases where visceral fat is responsible for the abnormally increased production of various proinflammatory adipokines including tumor necrosis factor-alpha (TNF-α), monocyte chemoattractant protein-1 (MCP-1), and interleukin 6 (IL-6) [121,122]. While WAT contains various other types of immune cells such as dendritic cells, T-lymphocytes, B-cells, and neutrophils during obesity, increased number of macrophages infiltrated into adipose tissue attracted by adipocyte-released chemokines as well as changes in inflammatory phenotype of residing macrophages induced by adipose hypertrophy, adipocyte necrosis, and increased lipotoxicity play a major role in initiating a low-grade systematic inflammation in obesity, which has been reviewed elsewhere [123]. 

Accumulating evidence shows that inflammation initiated from adipose tissue is one of the major contributing factors for the development of IR and T2D. In particular, TNF-α secreted from adipose tissue [124] and from the infiltrated macrophages in adipose tissue [125] may play an important role in developing obesity-associated IR. TNF-α expression levels are elevated in obese and diabetic rodent models [126]. Adipocytes exposed to TNF-α impaired insulin-stimulated glucose uptake via reducing insulin activation of IRS-1 [127], whereas neutralization of TNF-α significantly improved insulin-mediated peripheral glucose uptake [126]. Consistently, deletion of TNF-α gene in rodent models of obesity protected them from developing IR [128]. One of the proposed mechanisms by which TNF-α induces IR is through the activation of the c-Jun N-terminal kinase (JNK), and I kappa beta kinase (IκβK), which subsequently phosphorylates IRS-1 on Ser^307^, thereby suppressing IRS-1-mediated insulin action [129]. In addition, TNF-α inhibits the activity of AMP-activated protein kinase (AMPK), which is considered to be a master regulator of whole body energy homeostasis critical for maintaining insulin sensitivity [130]. Activation of AMPK inhibits hepatic gluconeogenesis [131], promotes fatty acids oxidation [132], inhibits fatty acid synthesis [133,134], regulates PGC-1α-mediated mitochondrial biogenesis [135,136], and increases GLUT4 expression in skeletal muscle [137]. It was demonstrated that the inhibition of AMPK in the muscle by TNF-α lead to the development of IR [138].

IL-6 is another cytokine that could be secreted from adipose tissue and may be associated with IR. IL-6 plasma levels were robustly elevated in obesity [139]. In T2D individuals, IL-6 was independently associated with IR and hyperglycemia [140]. It can induce hepatic production of the inflammatory marker C-reactive protein (CRP) [141], suggesting a role in IR [142]. However, it is still unclear whether IL-6 adversely affects glucose uptake and metabolism. In healthy mice and humans IL-6 can enhance fatty acid oxidation and an insulin-stimulated glucose uptake [143,144]. In T2D subjects, however, insulin-stimulated glucose uptake was not affected by intravenous infusion of IL-6 [145].

Adiponectin is a plasma protein secreted from mature adipocytes and has insulin-sensitizing effect [146]. Unlike other adipokines, circulating adiponectin concentrations are inversely associated with markers of IR and development of T2D [147,148]. Low levels of adiponectin found in obesity were associated with inflammation, whereas a loss in weight increased circulating adiponectin [149]. Adiponectin administration reversed IR in rodent models of obesity [150,151]. Adiponectin binds to its receptors adiponectin receptor protein 1(AdipoR1) and adiponectin receptor protein 2 (AdipoR2), which are expressed in the liver and skeletal muscle [152] and mediate its various biological effects, including activation of peroxisome proliferator-activated receptor (PPAR)-α [153] and AMPK [154]. Adiponectin may enhance insulin sensitivity via activating the AdipoR1/liver kinase B1 (LKB1)/AMPK pathway, which then suppresses the expression of sterol regulatory element-binding protein (SREBP)-1c [155]. SREBP-1c modulates fat metabolism by upregulating key enzymes on the hepatic fatty acid synthesis pathway such as fatty acid synthase, acetyl coenzyme A (acetyl-CoA) carboxylase, acetyl-CoA synthetase and others. Together, the suppression of SREBP1c and activation of AMPK improve fatty acid metabolism and prevent its accumulation in the liver thereby enhancing insulin sensitivity [156]. Adiponectin may also have functions independent of its receptors. Interestingly, one study showed that adiponectin enhanced insulin sensitivity in mice by inducing the production of IL-6 from macrophages, which then upregulated hepatic IRS-2 expression independent of adiponectin known receptors [157]. 

### 2.2. Impaired Insulin Secretion and Development of T2D

It is well known that T2D is characterized by impaired insulin secretion [158]. Both β-cell dysfunction and a decrease in β-cell mass contribute to insulin secretion abnormalities in T2D [159]. There are common mechanisms that regulate both β-cell insulin secretory function and mass [160], suggesting that a combination of β-cell dysfunction and loss of mass is the precipitating factor for impaired insulin secretion in T2D [161]. Progression in β-cell impairment may be at least partially due to increased production of ROS, which results from the abnormal antioxidant status in T2D [162,163]. The increased ROS production in β-cells could be generated from excess amounts of saturated fatty acids (lipotoxicity) and glucose (glucotoxicity) that gradually cause β-cell apoptosis and impair cellular function, thereby contributing to the pathogenesis of T2D [164,165,166,167].

## 3. Flavonoids

### 3.1. Discovery and Classifications

Flavonoids are widespread in the plant kingdom [168]. They are synthesized in plants as secondary metabolites from phenylalanine [169]. Many flavonoids have antioxidant capabilities [170] that protect plant membrane from desiccation, oxidation [171], and ultraviolet (UV) damage [172]. Flavonoids are important for the proper development of plants [173], and can improve plant growth [174] and support plant defense systems against microbial invasion [175].

Plants and food products containing flavonoids have been used to treat various human diseases since ancient times [176], although flavonoids were not discovered and characterized until the twentieth century. In the 1930s, Rusznyak and Szent-Gyorgyi extracted a substance containing a mixture of flavonoids from Hungarian peppers that had an action on vascular permeability and named it vitamin P [177]. The advances in flavonoids research in the 1970s led to the discovery of many other flavonoids, clearing the path for characterization of their structures and biological activities [178]. Although flavonoids were classified as semi-essential food components [179] and suggested as a new class of drugs due to their potential in treating many human diseases [168], extensive research on their effects on disease prevention was not started until the middle of the 1990s [180]. Recently, flavonoids have been referred to as nutraceuticals [181], a hybrid term describing a product that combines nutrients and pharmaceuticals [182] and defined as “any non-toxic food extract supplement that has scientifically proven health benefits for both the treatment and prevention of disease” [183].

**Figure 1:**
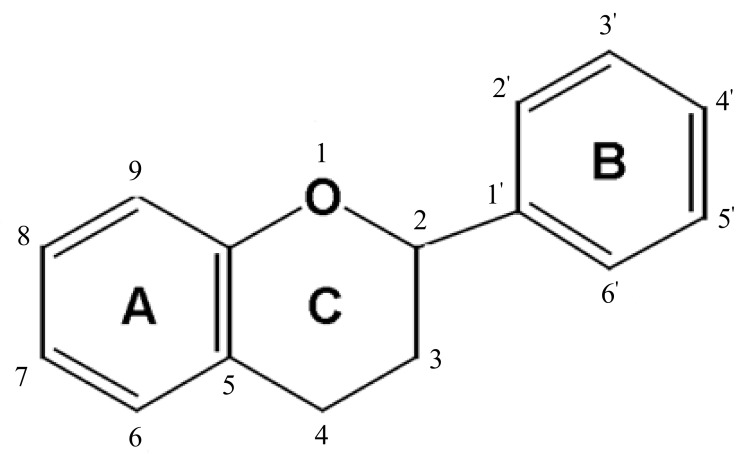
General structure of flavonoids.

Over 9000 flavonoid compounds have been identified from plant sources [184] sharing the basic chemical structure of a common three-ring moiety (A-, C- and B-rings) with 15 carbon atoms (C6–C3–C6) (Figure 1). The substitution of a functional group of the heterocyclic ring (C-ring) with a methyl, hydroxyl, glycan, acetyl or other group, along with the C-ring oxidation state determines the classification of various subclasses of flavonoids [185]. Flavonoids in each subclass are further structurally diversified due to different patterns of hydroxylation of the phenolic rings.

Flavonoids are divided into two main groups based on structure, 2-phenylchromans (flavonoids) and 3-phenylchromans (isoflavonoids). The 2-phenylchromans group includes the subclasses of flavanones, flavones, flavonols, flavan-3-ols, and anthocyanidins; whereas the 3-phenylchromans includes the subclasses of isoflavones, isoflavans, and pterocarpans [186]. 

The content of flavonoids in plants varies depending on many factors such as plant species, organ, stage of development, and environmental conditions [187]. In addition, food preparation and processing methods can significantly affect flavonoid content in foods [188,189]. Food databases provide information about food content of some flavonoids from six subclasses of flavonoids and with isoflavonoids separated from other subclasses [190]. According to the United States Department of Agriculture (USDA) database for the flavonoid content of selected foods (Table 1), the flavanones hesperetin, naringenin, and eriodictyol are primarily found in citrus fruits and their juices. Flavones luteolin and apigenin are predominant in aromatic herbs, parsley, celery, and peppers. Flavonols quercetin, kaempferol, myricetin, and isorhamnetin are found in many fruits and vegetables like apples, cranberries, onions, beans, and fennel. The flavan-3-ols (+)-catechin, (−)-epicatechin, (−)-epigallocatechin, theaflavin and their gallate esters are found in large amounts in tea, wine, and cocoa. Anthocyanidins cyanidin, delphinidin, malvidin, pelargonidin, peonidin, and petunidin are present in different varieties of berries, grapes, nuts and some vegetables. The USDA database for the isoflavone content of selected foods shows that the isoflavones daidzein, genistein, and glycitein are found in considerable quantities primarily in soybeans and soy products [191,192]. 

### 3.2. Dietary Intake

Several studies attempted to estimate the dietary intake of flavonoids in the U.S. It was estimated in one study that total dietary intake of flavonoids by U.S adults was 190 mg/day [193] while this number was 345 mg/day in another study [194]. These two studies analyzed 24-h dietary recall data from the National Health and Nutrition Examination Survey (NHANES) and used the USDA databases to assess the intake of flavonoids. However, the data used in these studies were from two different time periods, and the databases were from different releases and updates which may explain the difference in the estimated daily intake. Moreover, the highest intake of all flavonoids documented in these studies was from the subclass flavan-3-ols corresponding to a higher intake of tea [193,194]. Recently, USDA expanded flavonoids databases to include 2900 foods with analyzed contents of the five subclasses of flavonoids and the major isoflavones instead of the roughly 500 foods in the original [195]. This expansion is expected to provide researchers with a better tool to estimate dietary intake of flavonoids and study their effects on populations following exposure to these phytochemicals. Thus, re-analysis of the data from NHANES using the most recent USDA databases is recommended to achieve a more accurate assessment of intake. 

### 3.3. Absorption, Metabolism, and Bioavailability

How well flavonoids are absorbed and to what degree they are metabolized in the human body may determine their potential efficacy for the treatment and prevention of diseases. Starting in the mouth, flavonoids are first released from the plant matrix and some flavonoid glycosides (with sugar moiety) are hydrolyzed to aglycones (without sugar moiety) by saliva [196]. While some flavonoids are absorbed in the stomach [197], most of them undergo enzymatic hydrolysis and further metabolism in the small intestine [198,199]. The hydrolysis involves the deglycosylation of flavonoids, removal of glycosides naturally-bound to flavonoids by beta-glucosidases [200]. The hydrolyzed flavonoids are further metabolized by conjugation with glucuronic acid in the small intestine. The conjugation depends on the flavonoid structure with less predisposition to glucuronidation in flavonoids with a hydroxyl group on the B-ring [201], but more extensive metabolism and/or conjugation to flavan-3-ols [202]. The conjugation pattern is also affected by nutritional status. For example, administration of isoflavones in the fasting state results in more conjugation with sulfates and less glucuronidation than in the non-fasting state in humans [197]. Once absorbed, flavonoids from the small intestine reach the liver where they can be further conjugated with sulfate and/or methyl groups or excreted back with bile components [203,204,205].

The majority of ingested flavonoids may not undergo hydrolysis or conjugation in the small intestine [206], and are neither absorbed nor excreted from the bile [205]. Instead, these flavonoids pass to the colon where they are degraded by colonic flora into smaller molecules and phenolic acids that can then be absorbed [207,208].

The absorption of flavonoids depends on many factors such as the configuration of their structures and glycosylation [209]. In foods, flavonoids mostly exist in the glycosylated forms [210]. Some flavonoids can be absorbed more readily when attached to glycosides in the small intestine [211], while other flavonoids are absorbed more efficiently as aglycones [212,213]. In addition, the type of sugar moiety (galactose, rhamnose, arabinopyranose) of the glycoside [214], and the plant matrix can affect the absorption of flavonoids [215]. For instance, conjugation of the flavonol quercetin with glucose is associated with a greater absorption rate than the quercetin rutinoside irrespective of the glucose position on the quercetin molecule [216,217].

During the past two decades, research has primarily focused on exploring the potential biological and pharmacological functions of flavonoids such as the antioxidant [218], anti-inflammatory [219], and anticancer activities [220]. In this context, many factors that may affect their bioavailability should be taken into consideration to validate their health-promoting effects. Flavonoid bioavailability is influenced by many factors such as absorption rate, metabolism, conjugation, structure, and molecular weight [205,221]. In general, the bioavailability of flavonoids is low, and the majority of flavonoids are detected in the conjugated form in the plasma [222,223]. Among the various classes of flavonoids, isoflavones have the highest bioavailability, whereas anthocyanins have the lowest [224]. Ingestion of 50 mg anthocyanins as aglycone equivalents resulted in a maximal plasma concentration of about 30 nmol/L, while the plasma concentration reached 3 µmol/L after intake of the same amount of isoflavones [225]. Flavonoids can be detected in plasma after 30 min and cleared in several hours after ingestion [226,227], although the half-life for conjugated flavonoids could be as long as 28 h [228]. Other factors that may affect the bioavailability of flavonoids are variation in absorption and metabolism of flavonoids between individuals [229], flavonoid dose [230], and duration of consumption [231]. 

While conjugation of flavonoids is reported to alter their structure and likely reduce their biological activities [218,232], the microbial metabolism of flavonoids in the gut could generate a variety of metabolites with different biological activities [233]. Results from in vitro studies that explore biological roles of some flavonoids using their unconjugated forms should be analyzed with caution because they may not even be detectable in the plasma. More focus should be given to investigating flavonoid metabolites, which might be more bioavailable [229].

### 3.4. Potential Adverse Effects and Toxicity

Our daily diet contains considerable amounts of flavonoids, most of which are considered safe [179,234]. Galati and others reviewed the potential toxicity of flavonoids [235], and although one of the proposed actions of many flavonoids is antioxidant activity, in the presence of copper they can become pro-oxidants [218]. Similarly, long-term intake of quercetin may cause mutagenicity [236] and DNA damage that is further enhanced in the presence of copper, suggesting that mutagenesis caused by some flavonoids may be due to their pro-oxidant activity [237,238]. The use of supplements, including non-nutritive supplements, has been on the rise in recent years [239]; however, the purported benefits of flavonoid supplementation are not well justified by research results [240]. Thus, the use of flavonoids as dietary supplements in large quantities should not be encouraged until their biological effects are elucidated [241] and potential adverse effects are better understood [218,242].

## 4. Flavonoids and T2D

As discussed above, T2D is a result of chronic IR and loss of β-cell mass and function [243,244]. Thus, the search for agents that may promote insulin sensitivity and β-cell survival may provide a more effective strategy to prevent the onset of diabetes [245,246,247,248]. 

### 4.1. Flavonoids and the Prevention and Treatment of T2D 

#### 4.1.1. Antioxidant Activity of Flavonoids and T2D

One of the suggested triggers causing β-cell dysfunction and IR that ultimately lead to T2D is excessive ROS production [249], which may be due to the activation of stress signaling pathways [250]. Results from studies using cell cultures and animal models show that flavonoids can directly scavenge ROS [251,252]. Flavonoids can protect and restore antioxidant defense enzymes such as superoxide dismutase, catalase, and glutathione peroxidase [253,254], and inhibit ROS-producing enzymes such as xanthine oxidase [255]. As a consequence, flavonoids can inhibit several ROS-stimulated biological events such as inhibiting oxidized LDL (oxLDL)-induced cell apoptosis, and NF-κB-mediated transcriptional activity and subsequent inflammation [254]. In humans, the plasma concentrations of flavonoids after dietary intake are typically far less than those used in vitro studies for achieving strong scavenging capabilities, suggesting that these polyphenolic compounds may not have signifacnt antioxidant effect in vivo. Indeed, it was proposed that flavonoids themselves may only have minimal contribution to the antioxidant capacity in humans where the greatest contribution is from other components in flavonoid-rich foods [256]. However, there is the possibility that some flavonoids may exert antioxidant activities in vivo through modulating protein kinases that mediate ROS-induced signaling pathways [257]. Another possibility is that flavonoids might exert antioxidant effects and ROS scavenging capabilities in the digestive tract [258].

#### 4.1.2. Effects of Flavonoids on Postprandial Blood Glucose

The first step in carbohydrate digestion is their breakdown into absorbable monosaccharides in the small intestine. Carbohydrates are digested by enzymes such as α-amylase and α-glucosidase, and after that by small intestinal maltase, sucrase, and lactase [259]. As discussed above, glucose absorption in the small intestine is facilitated via carriers such as GLUT2 and SGLT1. The pharmacological inhibition of the digestive enzymes and/or glucose transporters will reduce glucose absorption, thereby lowering postprandial blood glucose levels [260,261]. The flavonols rutin, kaempferol, and quercetin inhibited carbohydrate digestion and absorption in experimental studies. Rutin inhibited α-glycosidase activity in vitro by directly binding to the enzyme through hydrophobic bonding [262]. In addition, kaempferol inhibited α-glycosidase activity in vitro [263]. Quercetin displayed more inhibitory activities through the inhibition of both maltase and sucrase activities in vitro and in vivo [264] and the suppression of SGLT1-mediated glucoside uptake in human intestinal Caco-2 cells via interaction with the transporter [265]. Also, quercetin inhibited GLUT2-mediated uptake in vitro (noncompetitive inhibition) and reduced postprandial blood glucose levels in diabetic mice when it was orally administered with glucose compared to glucose only administration [266]. Moreover, tiliroside a flavonol glycoside, reduced glucose absorption by competitive inhibition of the intestinal glucose transporter SGLT1 [267] and inhibited the elevation of blood glucose after an oral glucose load in IR animal model [267]. Flavonoid-rich extracts from Helichrysum and grapefruit inhibited the activity of α-amylase and α-glycosidase and suppressed SGLT1-mediated uptake in Caco-2 cells and lowered postprandial blood glucose after the oral administered accompanied with the ingestion of maltose or starch in healthy rats [268]. On the other hand, the flavone luteolin suppressed the activity of α-amylase and α-glycosidase in vitro [269], but had no significant effect on postprandial glucose levels in healthy rats even at a high dose (up to 200 mg/kg) [270].

Tea flavonoids, flavan-3-ols, such as catechin, epicatechin gallate and epigallocatechin gallate (EGCG) reduced glucose absorption by competitive inhibition of the intestinal glucose transporter SGLT1 in vitro [271,272] and showed some ability to delay intestinal absorption of glucose in healthy subjects [273]. Additionally, flavonoid extract from sugarcane reduced the glycemic response to a high glycemic meal in healthy subjects [274].

Proanthocyanin-rich extracts from raspberry and rowanberry inhibited α-amylase activity and acted synergistically with the drug acarbose to inhibit α-amylase activity in vitro [275]. Also, anthocyanins-rich extract from raspberry, blueberry, cranberry, strawberry, and other berries reduced glucose absorption by inhibiting SGLT1 and GUT2 and their expression in human intestinal Caco-2 cells [276]. Moreover, in experimental studies, the oral administration of a flavonoid-rich extract of serviceberry in diet-induced obese and hyperglycemic mice delayed carbohydrate absorption and subsequently ameliorated postprandial hyperglycemia at least partially through inhibiting intestinal α-glucosidase activity [277]. However, in clinical trials, the consumption of a variety of berries had different outcomes on glycemic response in healthy and T2D subjects. Consumption of a berry meal (containing bilberries, blackcurrants, cranberries, and strawberries) sweetened with sucrose, delayed glucose appearance in the blood of healthy subjects [278]. Similarly, in another study, sweetened blackcurrant juice fortified with crowberry powder improved postprandial glycemic control in healthy subjects [279]. On the contrary, the addition of raspberries and blueberries to starch-based food did not reduce blood glucose in healthy subjects [280]. The consumption of a sweetened cranberry juice did not improve the glycemic responses in healthy subjects, but some improvement was noted with unsweetened cranberry juice [281]. The latter finding was consistent with another study in which a cranberry product led to improvement in the glycemic response in T2D subjects when compared with cranberry products that contained more sugar [282].

Findings from the in vitro and animal studies regarding the modulatory effect of flavonoids on carbohydrate digestion and absorption thus postprandial blood glucose levels may have physiological relevance. While the inhibitory action on carbohydrate digestive enzymes of several flavonoids, flavones and flavonols, was suggested to be structure-related [283], some flavonoids such as the flavones from bamboo leaf extract, specifically vitexin, orientin, isovitexin, and isoorientin, inhibited starch digestion by interacting with the enzymes and with the starch molecule itself [284]. However, it is unclear in the clinical trials whether the observed beneficial effects by dietary consumption of flavonoids-rich products are partially or entirely ascribed to flavonoids. More effort should be directed at studying structure-function relationship to assist in predicting the efficacy of flavonoids in inhibiting enzymes involved in glucose digestion and absorption. In addition, long- and short-term studies to test different doses of flavonoids and products on glycemic responses in T2D subjects are needed.

#### 4.1.3. Effects of Flavonoids on Glucose Disposal

Another approach to preventing IR, hyperglycemia, T2D, and subsequent diabetic complications is to enhance glucose uptake by peripheral tissues. Various isoforms of glucose transporters (GLUT), are responsible for most glucose flux into cells [285]. GLUT4, the most abundant glucose transporter in both skeletal muscle and adipose tissue, is primarily regulated by insulin [286]. Insulin stimulation of glucose entry into cells is executed via inducing the translocation of GLUT4 to the plasma membranes of muscle and fat cells [287], which is promoted by a cascade of events including insulin signaling-triggered activation of the PI3K/Akt pathway [288]. In hepatocytes, glucose can be transported into and out of the cells by GLUT2 independent of insulin [289]. Additionally, activation of AMPK, a master regulator of energy metabolism, is considered to be one of the most important insulin-independent targets for improving glucose uptake in both muscle and adipose tissue [290,291]. 

Various flavonoids demonstrated capabilities of stimulating insulin-dependent and insulin-independent glucose uptake in peripheral tissues in vitro and in vivo. Procyanidins, polymers of the flavan-3-ols catechin and epicatechin, dose-dependently stimulated glucose uptake in L6E9 myotubes and 3T3-L1 adipocytes, which was associated with increased activity of Akt and extracellular signal–regulated kinase 1/2 (ERK1/2), another target of insulin signaling [292,293]. In vivo, the long-term provision of procyanidin extracted from grape seeds improved glucose homeostasis and insulin sensitivity in diet-induced hyperinsulinemic rats, effects that were consistent with the enhanced glucose uptake in cultured 3T3-L1 adipocytes [294]. However, the physiological relevance of these in vitro findings is unclear, because procyanidins might be difficult to be absorbed, given their oligomeric structures. In IR rats, treatment with green tea extract, mainly comprised of epicatechin, epigallocatechin, and their gallates, increased the expression of genes critical for glucose uptake and utilization such as Gsk3beta and Irs2 in the liver and Glut4 in the muscle [295]. Similarly, EGCG, the most abundant catechin in green tea, was shown to enhance insulin-stimulated glucose uptake by increasing GLUT4 membrane translocation in L6 myotubes. In addition, treatment with EGCG inhibited dexamethasone-induced IR via activating AMPK and Akt [296]. Similarly, the isoflavone genistein stimulated glucose uptake in L6 myotubes through activating AMPK and increasing the gene expression of GLUT4 and GLUT1 [297]. Whereas, the flavanone eriodictyol increased glucose uptake in hepatocytes and adipocytes under high-glucose conditions via activating the PI3K/Akt pathway [298]. Flavonoid 7-*O*-methylaromadendrin treatment stimulated glucose uptake in adipocytes by increasing the gene expression and activity of the transcriptional factor PPARγ2, and improved high glucose-induced IR in human hepatocellular liver carcinoma (HepG2) cells through activation of the PI3K/Akt and AMPK dependent pathways [299]. In addition, kaempferol and quercetin can also activate PPARγ and subsequently improve insulin-stimulated glucose uptake in 3T3-L1 adipocytes [300].

#### 4.1.4. Effects of Flavonoids on Obesity and Inflammation

Some flavonoids can improve dyslipidemia, modulate adipokine secretion, and inhibit adipogenesis, which can thereby ameliorate IR and T2D. Treatment with citrus flavonoids nobiletin and tangeretin increased adiponectin secretion in 3T3-L1 adipocytes while suppressing the production of monocyte chemoattractant protein-1 (MCP-1), a key chemokine involved in monocyte migration and infiltration into fat tissue [301]. Oral administration of naringin, a bioflavonoid from grapefruit, ameliorated dyslipidemia, hyperinsulinemia, hyperglycemia, hepatic steatosis, and IR in T2D rats, which were associated with reduced oxidative stress, upregulated PPARγ, reduced inflammatory markers, and improved β-cell function [302]. 

Treatment with flavonoid extracts from *Litsea coreana* (H.) Lev. reduced serum triglyceride, total cholesterol, and LDL-cholesterol, and improved insulin sensitivity in HF diet-fed IR hyperlipidemic rats [303,304]. Oral administration of tiliroside (100 mg/kg/day), a glycosidic flavonoid, ameliorated metabolic disorders in obese diabetic mice, which were associated with activation of multiple signaling molecules important for promoting energy metabolism and insulin sensitivity, including adiponectin, AMPK, and PPARα, in skeletal muscle and/or liver [305]. Tiliroside was found to inhibit α-amylase as well as SGLT1 and GLUT2 [267], suggesting that the observed metabolic effects of tiliroside could be partially mediated through inhibitory effects on intestinal carbohydrate digestion and glucose uptake. 

#### 4.1.5. Effect of Flavonoids on β-Cell Function

In IR, β-cells compensate for the defects in insulin action by releasing more insulin. T2D only develops when these cells are unable to secrete adequate amounts of insulin to compensate for the decreased insulin sensitivity. The decrease in insulin secretion is largely due to insulin secretory dysfunction and significant loss of functional β-cells [306,307,308,309,310]. Indeed, individuals with T2D always manifest increased β-cell apoptosis and reduced β-cell mass [308,309,311]. There are several proposed mechanisms underlying the β-cell dysfunction including increased generation of ROS, alterations in metabolic pathways, activation of endoplasmic reticulum stress, increases in intracellular calcium, among others [312].

Alloxan and streptozotocin (STZ) have been widely used to induce insulin-deficient diabetic animal models by selectively destroying β-cells [313]. In STZ-induced diabetic rats, intraperitoneal (ip) injection of quercetin improved glucose tolerance and dyslipidemia [314], effects that might be due to protection against β-cell apoptosis via a reduction in oxidative stress [315]. Similar results were observed with ip injection of naringenin 7-*O*-β-d-glucoside in STZ-induced diabetic rats [316]. Interestingly, treatment with epicatechin improved blood glucose by stimulating regeneration of functional pancreatic β-cells in alloxan-induced diabetic rats [317]. Epicatechin may also preserve β-cell mass and function through protection against oxidative stress [318]. Likewise, other flavonoids such as rutin and apigenin protected the islets against STZ-induced damage, probably due to their antioxidant activity [319]. 

The potential effects of dietary intake of isoflavones or soy products that are enriched with isoflavones, particularly genistein and daidzein, on diabetes have been extensively studied [320,321]. Dietary supplementation of genistein improved hyperglycemia, glucose tolerance, and blood insulin levels in various diabetic mouse models, including non-obese diabetic (NOD) mice [322,323] and STZ induced lean and obese diabetic mice [324,325], which were associated with improved β-cell proliferation and islet mass and insulin content. At the cellular levels, it has been demonstrated that genistein can directly act on pancreatic β-cells, inducing glucose-dependent insulin secretion and cell proliferation via activating the cAMP and ERK1/2 signaling pathways [324,326]. For more detailed information on the effects of isoflavones on β-cell function, please refer to this recent review article [327].

### 4.2. Flavonoid Intake and Risk of T2D in Humans 

A meta-analysis consisting of 6 cohort studies indicated that total flavonoid intake was associated with a reduced risk of T2D [328]. However, in an observational study, no association was found between dietary intake of flavonoids and the risk of T2D in postmenopausal women [329]. Interestingly, it was found that the flavonoid subclasses flavonols and flavanols are particularly associated with a lower risk T2D [330]. Specifically, there seems to be a reduced risk of T2D with greater intake of the flavonols quercetin and myricetin [331]. Data from several cohort studies demonstrated that tea, coffee, and their products which are rich in flavanols (Table 1), were associated with reduced risk of T2D [332,333,334,335]. Consistent with these results, a meta-analysis study concluded that consumption of 4 or more cups of tea derived from *Camellia sinensis* (L.) Kuntze per day may lower the risk of T2D [336]. In a human trial, long-term tea intake was associated with reduced fasting blood glucose and a lower risk of T2D [337]. Similarly, higher intake of anthocyanins or anthocyanin-rich plants like blueberries and grapes was also associated with a lower risk of T2D [338,339]. It should be noted that most of these studies used flavonoid-containing foods that also contain other phytochemicals, which could contribute to the observed health beneficial effects [340], possibly in a synergistic or additive manner [341,342,343]. Thus, it is possible that crude extracts or a combination of different pure compounds are more effective than isolated pure flavonoids at an equivalent dose for preventing and treating diabetes.

### 4.3. Effects of Flavonoids on T2D in Clinical Interventions

Results from clinical trials show different outcomes based on flavonoid subclasses. Supplementation with flavonoids such as silymarin [344] and silybin-beta-cyclodextrin [345] improved glycemic and lipidemic profiles in T2D subjects. Similarly, cranberry juice consumption for 3 months improved glycemic control in T2D subjects [346], where consumption of chokeberry juice for 3 months improved both the glycemic and lipidemic profiles in T2D subjects [347]. Supplementation with grape seed extract improved markers of inflammation and glycemic control in obese T2D subjects [348] and consumption of grapes for 3 weeks lowered plasma LDL-cholesterol and cholesterol in obese subjects [349]. Anthocyanin supplementation improved LDL- and HDL-cholesterol concentrations in dyslipidemic subjects [350] and reduced the inflammatory response in hypercholesterolemic subjects [351]. However, studies with tea catechins have yielded conflicting results. The consumption of green tea (456 mg catechins for 2 months or 9 g of green tea for 1 month) did not exert any beneficial effect in T2D subjects [352,353]. These outcomes were consistent with results from two other reports showing that dietary provision of green and black tea extract mixture (150 mg of green tea catechins and 75 mg of black tea theaflavin for 3 months) in T2D subjects [354] or green tea extract only (500 mg tea catechins for 4 months) in obese subjects with T2D [355]. However, one study found that intake of catechins (582.8 mg of catechins for 3 months) reduced the body weight of obese subjects with T2D, with some improvements in glucose control [356]. In line with this result, another study also showed that consumption of catechin-rich green tea (615 mg green tea catechins for 1 month) improved postprandial glucose in T2D subjects [357]. Collectively, it is still unclear whether tea catechins are effective in the treatment of T2D. Higher doses of tea extract and individual catechins such as EGCG, rather than a mixture, might be more effective in preventing or treating T2D in humans. In addition, the efficacy may depend on the metabolic state of the subjects and the duration of treatment.

## 5. Conclusions and Limitations

T2D is a progressive metabolic disorder that is increasing in prevalence globally and is a significant healthcare burden. Although the specific causes of T2D still need to be elucidated, there is a considerable body of evidence to support that IR and loss of functional β-cell mass play a major role in the etiology of the disease. Various mechanisms contribute to the development of IR and β-cell impairment and the magnitude of their effects is dependent on a combination of genetic and environmental factors. In the search for naturally occurring compounds to prevent and treat IR and T2D, flavonoids have drawn considerable attention for their potential anti-diabetic activities. Epidemiological studies indicate that higher intake of flavonoids is associated with reduced risk of T2D. Experimental studies have shown that flavonoids may reduce postprandial blood glucose by inhibiting glucose digestion and transport in the small intestine, by increasing glucose disposal in tissues, and by protecting and regenerating impaired β-cells, and/or enhancing pancreatic insulin secretion. As most of previously tested flavonoids only have moderate potency in preventing and treating diabetes, further investigation is needed to identify specific flavonoids with complementary roles in glucose metabolism, and further explore whether combinations of two or more flavonoids could be more effective in preventing and treating T2D.

As discussed above, rodent and in vitro models have been widely employed to determine the potential anti-diabetic mechanisms of flavonoids and flavonoids-enriched extracts. However, Ingested flavonoids from the diets in both humans and animals always undergo extensive transformation in the intestine and the liver [203,204,205], and thus the majority of flavonoids detected in the plasma (>90%) are in conjugated form [222,223], which are likely less biologically active [218,232]. Thus, the concentrations of the parent compounds in blood are only in the range of nanomoles to sub-micromoles following dietary supplementation. However, most in vitro studies that explored the fundamental roles of some flavonoids used the unconjugated forms, and the observed results were achieved at pharmacological doses (>10 µM) that are well above those physiologically attainable by dietary means (<5 µM). Therefore, the physiological relevance of these in vitro findings is still largely unknown. 

It was recently reported that microbiota composition is altered in patients with T2D [358,359,360], suggesting that gut microflora may play an important role in glucose hemostasis and pathogenesis of T2D [361]. Indeed, it was shown that increased *Bifidobacterium* spp. population in the gut is associated with improved glucose tolerance and insulin sensitivity while reduced inflammatory markers [362,363]. Gut microbiota also play a critical role in the biotransformation of flavonoids. The unabsorbed flavonoids in small intestine reach to the colon where they are metabolized by colonic microflora into smaller molecules such as phenolic acids [207,208], some of which might be more bioavailable and biologically active [229]. Meanwhile, some flavonoid metabolites in turn modulate the composition of microbiota by inhibiting the growth of pathogenic bacteria and promoting the growth of beneficial bacteria [364]. Therefore, the target for flavonoids (particularly those poorly absorbed flavonoids) to exert the antidiabetic effects might be restricted to the intestine partially via modulating intestinal microflora, an aspect that has been largely ignored during the past research. In future, it is also important to examine the antidiabetic effects of the major microbial metabolites of flavonoids using physiologically relevant concentrations found in the body. In that regard, it should be noted that there are differences in the intestinal bacterial species and activities for catalyzing and transforming flavonoids between rodent models and humans [365]. These differences need to be carefully considered for designing studies.

## Figures and Tables

**Table 1:** Major subclasses of flavonoids with examples and some of the major dietary sources.

Flavonoid Subclasses	Examples	Dietary Sources
Flavanones	Hesperetin, Naringenin, Eriodictyol, Naringin	Spice (dried oregano), grapefruit, lemon, orange, grapefruit juice, lemon juice, orange juice.
Flavones	Luteolin, Apigenin, Vitexin, Orientin	Spices (dried oregano, celery seed, dried parsley, thyme), celery, parsley, peppers.
Flavonols	Quercetin, Kaempferol, Myricetin, Isorhamnetin, Rutin, Tiliroside, Aromadendrin, Silymarin, Silybin	Capers, spice (saffron), apples, cranberries, arugula, asparagus, broccoli, cabbage, chives, coriander, endive, fennel, ginger, mustard greens, okra, onions, peppers, radish (raw, seeds, leaves), beans, buckwheat.
Flavan-3-ols	(+)-Catechin, (+)-Gallocatechin, (−)-Epicatechin, (−)-Epigallocatechin, (−)-Epicatechin 3-gallate, (−)-Epigallocatechin 3-gallate, Theaflavin, Theaflavin 3-gallate, Theaflavin 3′-gallate, Theaflavin 3,3′- digallate, Thearubigins	Apples, broad beans, pecans, pistachio, wine, cocoa, tea (green, black), soybeans.
Anthocyanidins	Cyanidin, Delphinidin, Malvidin, Pelargonidin, Peonidin, Petunidin	Berries (bilberry, blackberries, blackberry, chokeberry, elderberries, raspberries, blueberry, cranberry, serviceberry), currants, grapes, plum, red cabbage, eggplant, pecans, pistachio, wine, black beans.
Isoflavones	Daidzein, Genistein, Glycitein	Red clover, soybeans and soybean products (milk, flour, yogurt and others).

## References

[1] Centers of Disease Control and Prevention (2014). National Diabetes Statistics Report: Estimates of Diabetes and its Burden in the United States, 2014.

[2] Boyle J.P., Thompson T.J., Gregg E.W., Barker L.E., Williamson D.F. (2010). Projection of the year 2050 burden of diabetes in the us adult population: Dynamic modeling of incidence, mortality, and prediabetes prevalence. Popul. Health Metr..

[3] Zimmet P. (2003). The burden of type 2 diabetes: Are we doing enough?. Diabetes Metab..

[4] Assoc A.D. (2013). Economic costs of diabetes in the U.S. in 2012. Diabetes Care.

[5] American Diabetes Association (2010). Diagnosis and classification of diabetes mellitus. Diabetes Care.

[6] Goldberg I.J. (2001). Clinical review 124: Diabetic dyslipidemia: Causes and consequences. J. Clin. Endocrinol. Metab..

[7] Gougeon R., Pencharz P.B., Sigal R.J. (1997). Effect of glycemic control on the kinetics of whole-body protein metabolism in obese subjects with non-insulin-dependent diabetes mellitus during iso- and hypoenergetic feeding. Am. J. Clin. Nutr..

[8] Lewis G.F., Carpentier A., Adeli K., Giacca A. (2002). Disordered fat storage and mobilization in the pathogenesis of insulin resistance and type 2 diabetes. Endocr. Rev..

[9] Michael D.J., Ritzel R.A., Haataja L., Chow R.H. (2006). Pancreatic beta-cells secrete insulin in fast- and slow-release forms. Diabetes.

[10] Bergman R.N., Ader M., Huecking K., Van Citters G. (2002). Accurate assessment of beta-cell function: The hyperbolic correction. Diabetes.

[11] Ramlo-Halsted B.A., Edelman S.V. (2000). The natural history of type 2 diabetes: Practical points to consider in developing prevention and treatment strategies. Clin. Diabetes.

[12] Ahmed N. (2005). Advanced glycation endproducts—Role in pathology of diabetic complications. Diabetes Res. Clin. Pract..

[13] Stratton I.M., Adler A.I., Neil H.A., Matthews D.R., Manley S.E., Cull C.A., Hadden D., Turner R.C., Holman R.R. (2000). Association of glycaemia with macrovascular and microvascular complications of type 2 diabetes (ukpds 35): Prospective observational study. BMJ.

[14] Brownlee M. (2001). Biochemistry and molecular cell biology of diabetic complications. Nature.

[15] Rossetti L. (1995). Glucose toxicity: The implications of hyperglycemia in the pathophysiology of diabetes mellitus. Clin. Investig. Med..

[16] Ohkubo Y., Kishikawa H., Araki E., Miyata T., Isami S., Motoyoshi S., Kojima Y., Furuyoshi N., Shichiri M. (1995). Intensive insulin therapy prevents the progression of diabetic microvascular complications in japanese patients with non-insulin-dependent diabetes mellitus: A randomized prospective 6-year study. Diabetes Res. Clin. Pract..

[17] UK Prospective Diabetes Study (UKPDS) Group (1998). Intensive blood-glucose control with sulphonylureas or insulin compared with conventional treatment and risk of complications in patients with type 2 diabetes (UKPDS 33). Lancet.

[18] Nazimek-Siewniak B., Moczulski D., Grzeszczak W. (2002). Risk of macrovascular and microvascular complications in type 2 diabetes: Results of longitudinal study design. J. Diabetes Complicat..

[19] Holman R.R., Paul S.K., Bethel M.A., Matthews D.R., Neil H.A. (2008). 10-year follow-up of intensive glucose control in type 2 diabetes. N. Engl. J. Med..

[20] Groop L., Forsblom C., Lehtovirta M. (1997). Characterization of the prediabetic state. Am. J. Hypertens..

[21] Perfetti R., Barnett P.S., Mathur R., Egan J.M. (1998). Novel therapeutic strategies for the treatment of type 2 diabetes. Diabetes/Metab. Rev..

[22] Schulze M.B., Hu F.B. (2005). Primary prevention of diabetes: What can be done and how much can be prevented?. Annu. Rev. Publ Health.

[23] Keservani R., Sharma A. (2014). Flavonoids: Emerging trends and potential health benefits. J. Chin. Pharm. Sci..

[24] Triplitt C.L. (2012). Examining the mechanisms of glucose regulation. Am. J. Manag. Care.

[25] Roder P.V., Geillinger K.E., Zietek T.S., Thorens B., Koepsell H., Daniel H. (2014). The role of SGLT1 and GLUT2 in intestinal glucose transport and sensing. PLoS ONE.

[26] Agius L. (2008). Glucokinase and molecular aspects of liver glycogen metabolism. Biochem. J..

[27] Pagliassotti M.J., Cherrington A.D. (1992). Regulation of net hepatic glucose uptake in vivo. Annu. Rev. Physiol..

[28] Mithieux G. (1996). Role of glucokinase and glucose-6 phosphatase in the nutritional regulation of endogenous glucose production. Reprod. Nutr. Dev..

[29] Ferrannini E., Bjorkman O., Reichard G.A., Pilo A., Olsson M., Wahren J., DeFronzo R.A. (1985). The disposal of an oral glucose load in healthy subjects. A quantitative study. Diabetes.

[30] Efrat S., Tal M., Lodish H.F. (1994). The pancreatic beta-cell glucose sensor. Trends Biochem. Sci..

[31] Rutter G.A. (2001). Nutrient-secretion coupling in the pancreatic islet beta-cell: Recent advances. Mol. Aspects Med..

[32] Iezzi M., Kouri G., Fukuda M., Wollheim C.B. (2004). Synaptotagmin V and IX isoforms control Ca^2+^-dependent insulin exocytosis. J. Cell Sci..

[33] Newsholme P., Brennan L., Rubi B., Maechler P. (2005). New insights into amino acid metabolism, beta-cell function and diabetes. Clin. Sci..

[34] Edgerton D.S., Cardin S., Emshwiller M., Neal D., Chandramouli V., Schumann W.C., Landau B.R., Rossetti L., Cherrington A.D. (2001). Small increases in insulin inhibit hepatic glucose production solely caused by an effect on glycogen metabolism. Diabetes.

[35] Sindelar D.K., Chu C.A., Venson P., Donahue E.P., Neal D.W., Cherrington A.D. (1998). Basal hepatic glucose production is regulated by the portal vein insulin concentration. Diabetes.

[36] Capaldo B., Gastaldelli A., Antoniello S., Auletta M., Pardo F., Ciociaro D., Guida R., Ferrannini E., Sacca L. (1999). Splanchnic and leg substrate exchange after ingestion of a natural mixed meal in humans. Diabetes.

[37] Moore M.C., Cherrington A.D., Wasserman D.H. (2003). Regulation of hepatic and peripheral glucose disposal. Best Pract. Res. Clin. Endocrinol. Metab..

[38] Saccomani M.P., Bonadonna R.C., Bier D.M., DeFronzo R.A., Cobelli C. (1996). A model to measure insulin effects on glucose transport and phosphorylation in muscle: A three-tracer study. Am. J. Physiol..

[39] Kelley D., Mitrakou A., Marsh H., Schwenk F., Benn J., Sonnenberg G., Arcangeli M., Aoki T., Sorensen J., Berger M. (1988). Skeletal muscle glycolysis, oxidation, and storage of an oral glucose load. J. Clin. Investig..

[40] Taylor R., Price T.B., Katz L.D., Shulman R.G., Shulman G.I. (1993). Direct measurement of change in muscle glycogen concentration after a mixed meal in normal subjects. Am. J. Physiol..

[41] Jiang G., Zhang B.B. (2003). Glucagon and regulation of glucose metabolism. Am. J. Physiol. Endocrinol. Metab..

[42] Paolisso G., Scheen A.J., Albert A., Lefebvre P.J. (1989). Effects of pulsatile delivery of insulin and glucagon in humans. Am. J. Physiol..

[43] Klover P.J., Mooney R.A. (2004). Hepatocytes: Critical for glucose homeostasis. Int. J. Biochem. Cell Biol..

[44] Cherrington A.D. (1999). Banting lecture 1997. Control of glucose uptake and release by the liver in vivo. Diabetes.

[45] Nilsson L.H., Hultman E. (1974). Liver and muscle glycogen in man after glucose and fructose infusion. Scand. J. Clin. Lab. Investig..

[46] van Schaftingen E., Gerin I. (2002). The glucose-6-phosphatase system. Biochem. J..

[47] Maggs D.G., Jacob R., Rife F., Lange R., Leone P., During M.J., Tamborlane W.V., Sherwin R.S. (1995). Interstitial fluid concentrations of glycerol, glucose, and amino acids in human quadricep muscle and adipose tissue. Evidence for significant lipolysis in skeletal muscle. J. Clin. Investig..

[48] Saltiel A.R. (1996). Diverse signaling pathways in the cellular actions of insulin. Am. J. Physiol..

[49] Reaven G.M. (2005). The insulin resistance syndrome: Definition and dietary approaches to treatment. Annu. Rev. Nutr..

[50] Chung S.T., Hsia D.S., Chacko S.K., Rodriguez L.M., Haymond M.W. (2014). Increased gluconeogenesis in youth with newly diagnosed type 2 diabetes. Diabetologia.

[51] Bock G., Chittilapilly E., Basu R., Toffolo G., Cobelli C., Chandramouli V., Landau B.R., Rizza R.A. (2007). Contribution of hepatic and extrahepatic insulin resistance to the pathogenesis of impaired fasting glucose: Role of increased rates of gluconeogenesis. Diabetes.

[52] Basu R., Barosa C., Jones J., Dube S., Carter R., Basu A., Rizza R.A. (2013). Pathogenesis of prediabetes: Role of the liver in isolated fasting hyperglycemia and combined fasting and postprandial hyperglycemia. J. Clin. Endocrinol. Metab..

[53] Mykkanen L., Zaccaro D.J., Wagenknecht L.E., Robbins D.C., Gabriel M., Haffner S.M. (1998). Microalbuminuria is associated with insulin resistance in nondiabetic subjects: The insulin resistance atherosclerosis study. Diabetes.

[54] Triplitt C.L. (2012). Understanding the kidneys’ role in blood glucose regulation. Am. J. Manag. Care.

[55] Meyer C., Dostou J.M., Welle S.L., Gerich J.E. (2002). Role of human liver, kidney, and skeletal muscle in postprandial glucose homeostasis. Am. J. Physiol. Endocrinol. Metab..

[56] Meyer C., Dostou J.M., Gerich J.E. (1999). Role of the human kidney in glucose counterregulation. Diabetes.

[57] Gerich J.E. (2002). Hepatorenal glucose reciprocity in physiologic and pathologic conditions. Diabetes Nutr. Metab..

[58] Woerle H.J., Meyer C., Popa E.M., Cryer P.E., Gerich J.E. (2003). Renal compensation for impaired hepatic glucose release during hypoglycemia in type 2 diabetes: Further evidence for hepatorenal reciprocity. Diabetes.

[59] Marks J., Carvou N.J.C., Debnam E.S., Srai S.K., Unwin R.J. (2003). Diabetes increases facilitative glucose uptake and glut2 expression at the rat proximal tubule brush border membrane. J. Physiol..

[60] Noonan W.T., Shapiro V.M., Banks R.O. (2001). Renal glucose reabsorption during hypertonic glucose infusion in female streptozotocin-induced diabetic rats. Life Sci..

[61] Rahmoune H., Thompson P.W., Ward J.M., Smith C.D., Hong G., Brown J. (2005). Glucose transporters in human renal proximal tubular cells isolated from the urine of patients with non-insulin-dependent diabetes. Diabetes.

[62] Weickert M.O. (2012). Nutritional modulation of insulin resistance. Scientifica.

[63] Reaven G.M. (1988). Banting lecture 1988. Role of insulin resistance in human disease. Diabetes.

[64] Grundy S.M. (2006). Metabolic syndrome: Connecting and reconciling cardiovascular and diabetes worlds. J. Am. Coll. Cardiol..

[65] Lebovitz H.E. (2001). Insulin resistance: Definition and consequences. Exp. Clin. Endocrinol. Diabetes.

[66] Patti M.E. (1999). Nutrient modulation of cellular insulin action. Ann. N. Y. Acad. Sci..

[67] Krebs M., Roden M. (2004). Nutrient-induced insulin resistance in human skeletal muscle. Curr. Med. Chem..

[68] Lillioja S., Mott D.M., Spraul M., Ferraro R., Foley J.E., Ravussin E., Knowler W.C., Bennett P.H., Bogardus C. (1993). Insulin-resistance and insulin secretory dysfunction as precursors of non-insulin-dependent diabetes-mellitus—Prospective studies of pima-indians. N. Engl. J. Med..

[69] DeFronzo R.A., Tripathy D. (2009). Skeletal muscle insulin resistance is the primary defect in type 2 diabetes. Diabetes Care.

[70] Krook A., Bjornholm M., Galuska D., Jiang X.J., Fahlman R., Myers M.G., Wallberg-Henriksson H., Zierath J.R. (2000). Characterization of signal transduction and glucose transport in skeletal muscle from type 2 diabetic patients. Diabetes.

[71] Pratipanawatr W., Pratipanawatr T., Cusi K., Berria R., Adams J.M., Jenkinson C.P., Maezono K., DeFronzo R.A., Mandarino L.J. (2001). Skeletal muscle insulin resistance in normoglycemic subjects with a strong family history of type 2 diabetes is associated with decreased insulin-stimulated insulin receptor substrate-1 tyrosine phosphorylation. Diabetes.

[72] Cusi K., Maezono K., Osman A., Pendergrass M., Patti M.E., Pratipanawatr T., DeFronzo R.A., Kahn C.R., Mandarino L.J. (2000). Insulin resistance differentially affects the pi 3-kinase- and map kinase-mediated signaling in human muscle. J. Clin. Investig..

[73] Perseghin G., Price T.B., Petersen K.F., Roden M., Cline G.W., Gerow K., Rothman D.L., Shulman G.I. (1996). Increased glucose transport-phosphorylation and muscle glycogen synthesis after exercise training in insulin-resistant subjects. N. Engl. J. Med..

[74] Kahn B.B., Flier J.S. (2000). Obesity and insulin resistance. J. Clin. Investig..

[75] Itani S.I., Zhou Q., Pories W.J., MacDonald K.G., Dohm G.L. (2000). Involvement of protein kinase c in human skeletal muscle insulin resistance and obesity. Diabetes.

[76] Itani S.I., Ruderman N.B., Schmieder F., Boden G. (2002). Lipid-induced insulin resistance in human muscle is associated with changes in diacylglycerol, protein kinase C, and I kappa B-alpha. Diabetes.

[77] Lara-Castro C., Garvey W.T. (2008). Intracellular lipid accumulation in liver and muscle and the insulin resistance syndrome. Endocrinol. Metab. Clin. N. Am..

[78] Laybutt D.R., Schmitz-Peiffer C., Saha A.K., Ruderman N.B., Biden T.J., Kraegen E.W. (1999). Muscle lipid accumulation and protein kinase c activation in the insulin-resistant chronically glucose-infused rat. Am. J. Physiol..

[79] Pan D.A., Lillioja S., Kriketos A.D., Milner M.R., Baur L.A., Bogardus C., Jenkins A.B., Storlien L.H. (1997). Skeletal muscle triglyceride levels are inversely related to insulin action. Diabetes.

[80] Ikeda Y., Olsen G.S., Ziv E., Hansen L.L., Busch A.K., Hansen B.F., Shafrir E., Mosthaf-Seedorf L. (2001). Cellular mechanism of nutritionally induced insulin resistance in psammomys obesus: Overexpression of protein kinase cepsilon in skeletal muscle precedes the onset of hyperinsulinemia and hyperglycemia. Diabetes.

[81] Qu X., Seale J.P., Donnelly R. (1999). Tissue and isoform-selective activation of protein kinase c in insulin-resistant obese zucker rats-effects of feeding. J. Endocrinol..

[82] Mayer B., Oberbauer R. (2003). Mitochondrial regulation of apoptosis. News Physiol. Sci..

[83] Kelley D.E., Mokan M., Mandarino L.J. (1992). Intracellular defects in glucose metabolism in obese patients with niddm. Diabetes.

[84] Reaven G.M., Hollenbeck C., Jeng C.Y., Wu M.S., Chen Y.D. (1988). Measurement of plasma glucose, free fatty acid, lactate, and insulin for 24 h in patients with niddm. Diabetes.

[85] Petersen K.F., Dufour S., Shulman G.I. (2005). Decreased insulin-stimulated atp synthesis and phosphate transport in muscle of insulin-resistant offspring of type 2 diabetic parents. PLoS Med..

[86] Patti M.E., Butte A.J., Crunkhorn S., Cusi K., Berria R., Kashyap S., Miyazaki Y., Kohane I., Costello M., Saccone R. (2003). Coordinated reduction of genes of oxidative metabolism in humans with insulin resistance and diabetes: Potential role of PGC1 and NRF1. Proc. Natl. Acad. Sci. USA.

[87] Lefort N., Glancy B., Bowen B., Willis W.T., Bailowitz Z., De Filippis E.A., Brophy C., Meyer C., Hojlund K., Yi Z. (2010). Increased reactive oxygen species production and lower abundance of complex i subunits and carnitine palmitoyltransferase 1b protein despite normal mitochondrial respiration in insulin-resistant human skeletal muscle. Diabetes.

[88] Szendroedi J., Phielix E., Roden M. (2012). The role of mitochondria in insulin resistance and type 2 diabetes mellitus. Nat. Rev. Endocrinol..

[89] Hsueh W.A., Quinones M.J. (2003). Role of endothelial dysfunction in insulin resistance. Am. J. Cardiol..

[90] Pinkney J.H., Stehouwer C.D., Coppack S.W., Yudkin J.S. (1997). Endothelial dysfunction: Cause of the insulin resistance syndrome. Diabetes.

[91] Cersosimo E., DeFronzo R.A. (2006). Insulin resistance and endothelial dysfunction: The road map to cardiovascular diseases. Diabetes/Metab. Res. Rev..

[92] Rizza R.A. (2010). Pathogenesis of fasting and postprandial hyperglycemia in type 2 diabetes: Implications for therapy. Diabetes.

[93] Basu R., Chandramouli V., Dicke B., Landau B., Rizza R. (2005). Obesity and type 2 diabetes impair insulin-induced suppression of glycogenolysis as well as gluconeogenesis. Diabetes.

[94] Lin H.V., Accili D. (2011). Hormonal regulation of hepatic glucose production in health and disease. Cell Metab..

[95] Fisher S.J., Kahn C.R. (2003). Insulin signaling is required for insulin’s direct and indirect action on hepatic glucose production. J. Clin. Investig..

[96] Ishihara H., Maechler P., Gjinovci A., Herrera P.L., Wollheim C.B. (2003). Islet beta-cell secretion determines glucagon release from neighbouring alpha-cells. Nat. Cell Biol..

[97] Granner D., Andreone T., Sasaki K., Beale E. (1983). Inhibition of transcription of the phosphoenolpyruvate carboxykinase gene by insulin. Nature.

[98] Dickens M., Svitek C.A., Culbert A.A., O’Brien R.M., Tavare J.M. (1998). Central role for phosphatidylinositide 3-kinase in the repression of glucose-6-phosphatase gene transcription by insulin. J. Biol. Chem..

[99] Li X., Monks B., Ge Q., Birnbaum M.J. (2007). Akt/PKB regulates hepatic metabolism by directly inhibiting pgc-1alpha transcription coactivator. Nature.

[100] Cross D.A.E., Alessi D.R., Cohen P., Andjelkovich M., Hemmings B.A. (1995). Inhibition of glycogen-synthase kinase-3 by insulin-mediated by protein-kinase-b. Nature.

[101] van Weeren P.C., de Bruyn K.M.T., de Vries-Smits A.M.M., van Lint J., Burgering B.M.T. (1998). Essential role for protein kinase B (PKB) in insulin-induced glycogen synthase kinase 3 inactivation—Characterization of dominant-negative mutant of pkb. J. Biol. Chem..

[102] Arden K.C., Biggs W.H. (2002). Regulation of the foxo family of transcription factors by phosphatidylinositol-3 kinase-activated signaling. Arch. Biochem. Biophys..

[103] Samuel V.T., Beddow S.A., Iwasaki T., Zhang X.M., Chu X., Still C.D., Gerhard G.S., Shulman G.I. (2009). Fasting hyperglycemia is not associated with increased expression of PEPCK or G6Pc in patients with type 2 diabetes. Proc. Natl. Acad. Sci. USA.

[104] Clore J.N., Stillman J., Sugerman H. (2000). Glucose-6-phosphatase flux in vitro is increased in type 2 diabetes. Diabetes.

[105] Barthel A., Schmoll D., Unterman T.G. (2005). Foxo proteins in insulin action and metabolism. Trends Endocrinol. Metab..

[106] Zhang W., Patil S., Chauhan B., Guo S., Powell D.R., Le J., Klotsas A., Matika R., Xiao X., Franks R. (2006). Foxo1 regulates multiple metabolic pathways in the liver: Effects on gluconeogenic, glycolytic, and lipogenic gene expression. J. Biol. Chem..

[107] Haeusler R.A., Kaestner K.H., Accili D. (2010). Foxos function synergistically to promote glucose production. J. Biol. Chem..

[108] Puigserver P., Rhee J., Donovan J., Walkey C.J., Yoon J.C., Oriente F., Kitamura Y., Altomonte J., Dong H.J., Accili D. (2003). Insulin-regulated hepatic gluconeogenesis through FOXO1-PGC-1 alpha interaction. Nature.

[109] Wang M., Wang X.C., Zhang Z.Y., Mou B., Hu R.M. (2010). Impaired mitochondrial oxidative phosphorylation in multiple insulin-sensitive tissues of humans with type 2 diabetes mellitus. J. Int. Med. Res..

[110] Kim D.H., Perdomo G., Zhang T., Slusher S., Lee S., Phillips B.E., Fan Y., Giannoukakis N., Gramignoli R., Strom S. (2011). Foxo6 integrates insulin signaling with gluconeogenesis in the liver. Diabetes.

[111] Perry R.J., Samuel V.T., Petersen K.F., Shulman G.I. (2014). The role of hepatic lipids in hepatic insulin resistance and type 2 diabetes. Nature.

[112] Abdelmalek M.F., Diehl A.M. (2007). Nonalcoholic fatty liver disease as a complication of insulin resistance. Med. Clin. N. Am..

[113] Brons C., Jensen C.B., Storgaard H., Hiscock N.J., White A., Appel J.S., Jacobsen S., Nilsson E., Larsen C.M., Astrup A. (2009). Impact of short-term high-fat feeding on glucose and insulin metabolism in young healthy men. J. Physiol..

[114] Samuel V.T., Liu Z.X., Qu X., Elder B.D., Bilz S., Befroy D., Romanelli A.J., Shulman G.I. (2004). Mechanism of hepatic insulin resistance in non-alcoholic fatty liver disease. J. Biol. Chem..

[115] Magkos F., Su X., Bradley D., Fabbrini E., Conte C., Eagon J.C., Varela J.E., Brunt E.M., Patterson B.W., Klein S. (2012). Intrahepatic diacylglycerol content is associated with hepatic insulin resistance in obese subjects. Gastroenterology.

[116] Mantena S.K., Vaughn D.P., Andringa K.K., Eccleston H.B., King A.L., Abrams G.A., Doeller J.E., Kraus D.W., Darley-Usmar V.M., Bailey S.M. (2009). High fat diet induces dysregulation of hepatic oxygen gradients and mitochondrial function in vivo. Biochem. J..

[117] Seidell J.C. (2000). Obesity, insulin resistance and diabetes—A worldwide epidemic. Br. J. Nutr..

[118] Colditz G.A., Willett W.C., Stampfer M.J., Manson J.E., Hennekens C.H., Arky R.A., Speizer F.E. (1990). Weight as a risk factor for clinical diabetes in women. Am. J. Epidemiol..

[119] Xu H., Barnes G.T., Yang Q., Tan G., Yang D., Chou C.J., Sole J., Nichols A., Ross J.S., Tartaglia L.A. (2003). Chronic inflammation in fat plays a crucial role in the development of obesity-related insulin resistance. J. Clin. Investig..

[120] Berg A.H., Scherer P.E. (2005). Adipose tissue, inflammation, and cardiovascular disease. Circ. Res..

[121] Ferroni P., Basili S., Falco A., Davi G. (2004). Inflammation, insulin resistance, and obesity. Curr. Atheroscler. Rep..

[122] Permana P.A., Menge C., Reaven P.D. (2006). Macrophage-secreted factors induce adipocyte inflammation and insulin resistance. Biochem. Biophys. Res. Commun..

[123] Boutens L., Stienstra R. (2016). Adipose tissue macrophages: Going off track during obesity. Diabetologia.

[124] Tsigos C., Kyrou I., Chala E., Tsapogas P., Stavridis J.C., Raptis S.A., Katsilambros N. (1999). Circulating tumor necrosis factor alpha concentrations are higher in abdominal versus peripheral obesity. Metab. Clin. Exp..

[125] Weisberg S.P., McCann D., Desai M., Rosenbaum M., Leibel R.L., Ferrante A.W. (2003). Obesity is associated with macrophage accumulation in adipose tissue. J. Clin. Investig..

[126] Hotamisligil G.S., Shargill N.S., Spiegelman B.M. (1993). Adipose expression of tumor necrosis factor-alpha: Direct role in obesity-linked insulin resistance. Science.

[127] Hotamisligil G.S., Murray D.L., Choy L.N., Spiegelman B.M. (1994). Tumor necrosis factor alpha inhibits signaling from the insulin receptor. Proc. Natl. Acad. Sci. USA.

[128] Uysal K.T., Wiesbrock S.M., Marino M.W., Hotamisligil G.S. (1997). Protection from obesity-induced insulin resistance in mice lacking tnf-alpha function. Nature.

[129] Hotamisligil G.S. (2003). Inflammatory pathways and insulin action. Int. J. Obes. Relat. Metab. Disord..

[130] Steinberg G.R. (2007). Inflammation in obesity is the common link between defects in fatty acid metabolism and insulin resistance. Cell Cycle.

[131] Bergeron R., Previs S.F., Cline G.W., Perret P., Russell R.R., Young L.H., Shulman G.I. (2001). Effect of 5-aminoimidazole-4-carboxamide-1-beta-D-ribofuranoside infusion on in vivo glucose and lipid metabolism in lean and obese zucker rats. Diabetes.

[132] Steinberg G.R., Kemp B.E. (2009). AMPK in health and disease. Physiol. Rev..

[133] Carling D., Sanders M.J., Woods A. (2008). The regulation of amp-activated protein kinase by upstream kinases. Int. J. Obes..

[134] Witczak C.A., Sharoff C.G., Goodyear L.J. (2008). Amp-activated protein kinase in skeletal muscle: From structure and localization to its role as a master regulator of cellular metabolism. Cell. Mol. Life Sci..

[135] Jorgensen S.B., Wojtaszewski J.F., Viollet B., Andreelli F., Birk J.B., Hellsten Y., Schjerling P., Vaulont S., Neufer P.D., Richter E.A. (2005). Effects of alpha-AMPK knockout on exercise-induced gene activation in mouse skeletal muscle. FASEB J..

[136] Canto C., Gerhart-Hines Z., Feige J.N., Lagouge M., Noriega L., Milne J.C., Elliott P.J., Puigserver P., Auwerx J. (2009). AMPK regulates energy expenditure by modulating nad+ metabolism and sirt1 activity. Nature.

[137] Holmes B.F., Kurth-Kraczek E.J., Winder W.W. (1999). Chronic activation of 5′-AMP-activated protein kinase increases GLUT-4, hexokinase, and glycogen in muscle. J. Appl. Physiol..

[138] Steinberg G.R., Michell B.J., van Denderen B.J., Watt M.J., Carey A.L., Fam B.C., Andrikopoulos S., Proietto J., Gorgun C.Z., Carling D. (2006). Tumor necrosis factor alpha-induced skeletal muscle insulin resistance involves suppression of amp-kinase signaling. Cell Metab..

[139] Mohamed-Ali V., Goodrick S., Bulmer K., Holly J.M., Yudkin J.S., Coppack S.W. (1999). Production of soluble tumor necrosis factor receptors by human subcutaneous adipose tissue in vivo. Am. J. Physiol..

[140] Natali A., Toschi E., Baldeweg S., Ciociaro D., Favilla S., Sacca L., Ferrannini E. (2006). Clustering of insulin resistance with vascular dysfunction and low-grade inflammation in type 2 diabetes. Diabetes.

[141] Bataille R., Klein B. (1992). C-reactive protein-levels as a direct indicator of interleukin-6 levels in humans in vivo. Arthritis Rheum..

[142] Yudkin J.S., Stehouwer C.D., Emeis J.J., Coppack S.W. (1999). C-reactive protein in healthy subjects: Associations with obesity, insulin resistance, and endothelial dysfunction: A potential role for cytokines originating from adipose tissue?. Arterioscler. Thromb. Vasc. Biol..

[143] Carey A.L., Steinberg G.R., Macaulay S.L., Thomas W.G., Holmes A.G., Ramm G., Prelovsek O., Hohnen-Behrens C., Watt M.J., James D.E. (2006). Interleukin-6 increases insulin-stimulated glucose disposal in humans and glucose uptake and fatty acid oxidation in vitro via amp-activated protein kinase. Diabetes.

[144] Yuen D.Y., Dwyer R.M., Matthews V.B., Zhang L., Drew B.G., Neill B., Kingwell B.A., Clark M.G., Rattigan S., Febbraio M.A. (2009). Interleukin-6 attenuates insulin-mediated increases in endothelial cell signaling but augments skeletal muscle insulin action via differential effects on tumor necrosis factor-alpha expression. Diabetes.

[145] Harder-Lauridsen N.M., Krogh-Madsen R., Holst J.J., Plomgaard P., Leick L., Pedersen B.K., Fischer C.P. (2014). Effect of IL-6 on the insulin sensitivity in patients with type 2 diabetes. Am. J. Physiol. Endocrinol. Metab..

[146] Hu E., Liang P., Spiegelman B.M. (1996). Adipoq is a novel adipose-specific gene dysregulated in obesity. J. Biol. Chem..

[147] Fargnoli J.L., Sun Q., Olenczuk D., Qi L., Zhu Y., Hu F.B., Mantzoros C.S. (2010). Resistin is associated with biomarkers of inflammation while total and high-molecular weight adiponectin are associated with biomarkers of inflammation, insulin resistance, and endothelial function. Eur. J. Endocrinol..

[148] Swarbrick M.M., Havel P.J. (2008). Physiological, pharmacological, and nutritional regulation of circulating adiponectin concentrations in humans. Metab. Syndr. Relat. Disord..

[149] Kopp H.P., Krzyzanowska K., Mohlig M., Spranger J., Pfeiffer A.F., Schernthaner G. (2005). Effects of marked weight loss on plasma levels of adiponectin, markers of chronic subclinical inflammation and insulin resistance in morbidly obese women. Int. J. Obes..

[150] Yamauchi T., Kamon J., Waki H., Terauchi Y., Kubota N., Hara K., Mori Y., Ide T., Murakami K., Tsuboyama-Kasaoka N. (2001). The fat-derived hormone adiponectin reverses insulin resistance associated with both lipoatrophy and obesity. Nat. Med..

[151] Koch C.E., Lowe C., Legler K., Benzler J., Boucsein A., Bottiger G., Grattan D.R., Williams L.M., Tups A. (2014). Central adiponectin acutely improves glucose tolerance in male mice. Endocrinology.

[152] Yamauchi T., Kamon J., Ito Y., Tsuchida A., Yokomizo T., Kita S., Sugiyama T., Miyagishi M., Hara K., Tsunoda M. (2003). Cloning of adiponectin receptors that mediate antidiabetic metabolic effects. Nature.

[153] Yamauchi T., Nio Y., Maki T., Kobayashi M., Takazawa T., Iwabu M., Okada-Iwabu M., Kawamoto S., Kubota N., Kubota T. (2007). Targeted disruption of adipor1 and adipor2 causes abrogation of adiponectin binding and metabolic actions. Nat. Med..

[154] Yadav A., Kataria M.A., Saini V., Yadav A. (2013). Role of leptin and adiponectin in insulin resistance. Clin. Chim. Acta Int. J. Clin. Chem..

[155] Awazawa M., Ueki K., Inabe K., Yamauchi T., Kaneko K., Okazaki Y., Bardeesy N., Ohnishi S., Nagai R., Kadowaki T. (2009). Adiponectin suppresses hepatic srebp1c expression in an adipor1/lkb1/AMPK dependent pathway. Biochem. Biophys. Res. Commun..

[156] Yamauchi T., Kamon J., Minokoshi Y., Ito Y., Waki H., Uchida S., Yamashita S., Noda M., Kita S., Ueki K. (2002). Adiponectin stimulates glucose utilization and fatty-acid oxidation by activating amp-activated protein kinase. Nat. Med..

[157] Awazawa M., Ueki K., Inabe K., Yamauchi T., Kubota N., Kaneko K., Kobayashi M., Iwane A., Sasako T., Okazaki Y. (2011). Adiponectin enhances insulin sensitivity by increasing hepatic irs-2 expression via a macrophage-derived IL-6-dependent pathway. Cell Metab..

[158] Clark A., Jones L.C., de Koning E., Hansen B.C., Matthews D.R. (2001). Decreased insulin secretion in type 2 diabetes: A problem of cellular mass or function?. Diabetes.

[159] Kahn S.E., Zraika S., Utzschneider K.M., Hull R.L. (2009). The beta cell lesion in type 2 diabetes: There has to be a primary functional abnormality. Diabetologia.

[160] Donath M.Y., Ehses J.A., Maedler K., Schumann D.M., Ellingsgaard H., Eppler E., Reinecke M. (2005). Mechanisms of beta-cell death in type 2 diabetes. Diabetes.

[161] Meier J.J., Bonadonna R.C. (2013). Role of reduced beta-cell mass versus impaired beta-cell function in the pathogenesis of type 2 diabetes. Diabetes Care.

[162] Vijayalingam S., Parthiban A., Shanmugasundaram K.R., Mohan V. (1996). Abnormal antioxidant status in impaired glucose tolerance and non-insulin-dependent diabetes mellitus. Diabet. Med. J. Br. Diabet. Assoc..

[163] Bast A., Wolf G., Oberbaumer I., Walther R. (2002). Oxidative and nitrosative stress induces peroxiredoxins in pancreatic beta cells. Diabetologia.

[164] Robertson R.P., Harmon J., Tran P.O., Tanaka Y., Takahashi H. (2003). Glucose toxicity in beta-cells: Type 2 diabetes, good radicals gone bad, and the glutathione connection. Diabetes.

[165] Kashyap S., Belfort R., Gastaldelli A., Pratipanawatr T., Berria R., Pratipanawatr W., Bajaj M., Mandarino L., DeFronzo R., Cusi K. (2003). A sustained increase in plasma free fatty acids impairs insulin secretion in nondiabetic subjects genetically predisposed to develop type 2 diabetes. Diabetes.

[166] Santomauro A.T., Boden G., Silva M.E., Rocha D.M., Santos R.F., Ursich M.J., Strassmann P.G., Wajchenberg B.L. (1999). Overnight lowering of free fatty acids with acipimox improves insulin resistance and glucose tolerance in obese diabetic and nondiabetic subjects. Diabetes.

[167] Poitout V., Robertson R.P. (2002). Minireview: Secondary beta-cell failure in type 2 diabetes—A convergence of glucotoxicity and lipotoxicity. Endocrinology.

[168] Havsteen B. (1983). Flavonoids, a class of natural products of high pharmacological potency. Biochem. Pharmacol..

[169] Harborne J.B. (1986). Plant Flavonoids in Biology and Medicine.

[170] Stevenson D.E., Hurst R.D. (2007). Polyphenolic phytochemicals—Just antioxidants or much more?. Cell. Mol. Life Sci..

[171] Moore J.P., Westall K.L., Ravenscroft N., Farrant J.M., Lindsey G.G., Brandt W.F. (2005). The predominant polyphenol in the leaves of the resurrection plant myrothamnus flabellifolius, 3,4,5 tri-O-galloylquinic acid, protects membranes against desiccation and free radical-induced oxidation. Biochem. J..

[172] Ryan K.G., Swinny E.E., Markham K.R., Winefield C. (2002). Flavonoid gene expression and uv photoprotection in transgenic and mutant petunia leaves. Phytochemistry.

[173] Petrussa E., Braidot E., Zancani M., Peresson C., Bertolini A., Patui S., Vianello A. (2013). Plant flavonoids—Biosynthesis, transport and involvement in stress responses. Int. J. Mol. Sci..

[174] Webster G., Jain V., Davey M.R., Gough C., Vasse J., Denarie J., Cocking E.C. (1998). The flavonoid naringenin stimulates the intercellular colonization of wheat roots by azorhizobium caulinodans. Plant Cell Environ..

[175] Harborne J.B. (1999). The comparative biochemistry of phytoalexin induction in plants. Biochem. Syst. Ecol..

[176] Havsteen B.H. (2002). The biochemistry and medical significance of the flavonoids. Pharm. Ther..

[177] Rusznyak S., Szent-Gyorgyi A. (1936). Vitamin P: Flavonols as vitamins. Nature.

[178] Harborne J.B., Mabry T.J., Mabry H. (1975). The Flavonoids.

[179] Kuhnau J. (1976). The flavonoids. A class of semi-essential food components: Their role in human nutrition. World Rev. Nutr. Diet..

[180] Scalbert A., Johnson I.T., Saltmarsh M. (2005). Polyphenols: Antioxidants and beyond. Am. J. Clin. Nutr..

[181] Tapas A.R., Sakarkar D.M., Kakde R.B. (2008). Flavonoids as nutraceuticals: A review. Trop. J. Pharm. Res..

[182] Espin J.C., Garcia-Conesa M.T., Tomas-Barberan F.A. (2007). Nutraceuticals: Facts and fiction. Phytochemistry.

[183] Dillard C.J., German J.B. (2000). Phytochemicals: Nutraceuticals and human health. J. Sci. Food. Agric..

[184] Williams C.A., Grayer R.J. (2004). Anthocyanins and other flavonoids. Nat. Prod. Rep..

[185] Beecher G.R. (2003). Overview of dietary flavonoids: Nomenclature, occurrence and intake. J. Nutr..

[186] Dixon R.A., Pasinetti G.M. (2010). Flavonoids and isoflavonoids: From plant biology to agriculture and neuroscience. Plant Physiol..

[187] Debeaujon I., Peeters A.J., Leon-Kloosterziel K.M., Koornneef M. (2001). The transparent testa12 gene of arabidopsis encodes a multidrug secondary transporter-like protein required for flavonoid sequestration in vacuoles of the seed coat endothelium. Plant Cell.

[188] Hertog M.G.L., Hollman P.C.H., Katan M.B. (1992). Content of potentially anticarcinogenic flavonoids of 28 vegetables and 9 fruits commonly consumed in the netherlands. J. Agric. Food Chem..

[189] Ewald C., Fjelkner-Modig S., Johansson K., Sjoholm I., Akesson B. (1999). Effect of processing on major flavonoids in processed onions, green beans, and peas. Food Chem..

[190] Pennington J.A.T. (2002). Food composition databases for bioactive food components. J. Food. Compos. Anal..

[191] Bhagwat S., Haytowitz D.B., Holden J.M. (2013). USDA Database for the Flavonoid Content of Selected Foods, Release 3.1.

[192] Bhagwat S., Haytowitz D.B., Holden J.M. (2008). USDA Database for the Isoflavone Content of Selected Foods, Release 2.0.

[193] Chun O.K., Chung S.J., Song W.O. (2007). Estimated dietary flavonoid intake and major food sources of U.S. Adults. J. Nutr..

[194] Bai W., Wang C., Ren C. (2014). Intakes of total and individual flavonoids by us adults. Int. J. Food Sci. Nutr..

[195] Bhagwat S., Haytowitz D.B., Wasswa-Kintu S.I., Holden J.M. (2013). Usda develops a database for flavonoids to assess dietary intakes. Proc. Food Sci..

[196] Walle T., Browning A.M., Steed L.L., Reed S.G., Walle U.K. (2005). Flavonoid glucosides are hydrolyzed and thus activated in the oral cavity in humans. J. Nutr..

[197] Piskula M.K. (2000). Factors affecting flavonoids absorption. Biofactors.

[198] Walle T., Otake Y., Walle U.K., Wilson F.A. (2000). Quercetin glucosides are completely hydrolyzed in ileostomy patients before absorption. J. Nutr..

[199] Day A.J., DuPont M.S., Ridley S., Rhodes M., Rhodes M.J.C., Morgan M.R.A., Williamson G. (1998). Deglycosylation of flavonoid and isoflavonoid glycosides by human small intestine and liver beta-glucosidase activity. FEBS Lett..

[200] Nemeth K., Plumb G.W., Berrin J.G., Juge N., Jacob R., Naim H.Y., Williamson G., Swallow D.M., Kroon P.A. (2003). Deglycosylation by small intestinal epithelial cell beta-glucosidases is a critical step in the absorption and metabolism of dietary flavonoid glycosides in humans. Eur. J. Nutr..

[201] Spencer J.P.E., Chowrimootoo G., Choudhury R., Debnam E.S., Srai S.K., Rice-Evans C. (1999). The small intestine can both absorb and glucuronidate luminal flavonoids. FEBS Lett..

[202] Spencer J.P.E., Schroeter H., Rechner A.R., Rice-Evans C. (2001). Bioavailability of flavan-3-ols and procyanidins: Gastrointestinal tract influences and their relevance to bioactive forms in vivo. Antioxid. Redox Signal..

[203] Piskula M.K., Terao J. (1998). Accumulation of (-)-epicatechin metabolites in rat plasma after oral administration and distribution of conjugation enzymes in rat tissues. J. Nutr..

[204] Manach C., Morand C., Texier O., Favier M.L., Agullo G., Demigne C., Regerat F., Remesy C. (1995). Quercetin metabolites in plasma of rats fed diets containing rutin or quercetin. J. Nutr..

[205] Landete J.M. (2012). Updated knowledge about polyphenols: Functions, bioavailability, metabolism, and health. Crit. Rev. Food Sci. Nutr..

[206] Crespy V., Morand C., Manach C., Besson C., Demigne C., Remesy C. (1999). Part of quercetin absorbed in the small intestine is conjugated and further secreted in the intestinal lumen. Am. J. Physiol..

[207] Kim D.H., Jung E.A., Sohng I.S., Han J.A., Kim T.H., Han M.J. (1998). Intestinal bacterial metabolism of flavonoids and its relation to some biological activities. Arch. Pharm. Res..

[208] Meselhy M.R., Nakamura N., Hattori M. (1997). Biotransformation of (-)-epicatechin 3-o-gallate by human intestinal bacteria. Chem. Pharm. Bull..

[209] Scalbert A., Williamson G. (2000). Dietary intake and bioavailability of polyphenols. J. Nutr..

[210] Vukics V., Guttman A. (2010). Structural characterization of flavonoid glycosides by multi-stage mass spectrometry. Mass Spectrom. Rev..

[211] Hollman P.C., Katan M.B. (1999). Dietary flavonoids: Intake, health effects and bioavailability. Food Chem. Toxicol..

[212] Okushio K., Matsumoto N., Kohri T., Suzuki M., Nanjo F., Hara Y. (1996). Absorption of tea catechins into rat portal vein. Biol. Pharm. Bull..

[213] Crespy V., Morand C., Besson C., Manach C., Demigne C., Remesy C. (2002). Quercetin, but not its glycosides, is absorbed from the rat stomach. J. Agric. Food Chem..

[214] Arts I.C.W., Sesink A.L.A., Faassen-Peters M., Hollman P.C.H. (2004). The type of sugar moiety is a major determinant of the small intestinal uptake and subsequent biliary excretion of dietary quercetin glycosides. Br. J. Nutr..

[215] Graefe E.U., Wittig J., Mueller S., Riethling A.K., Uehleke B., Drewelow B., Pforte H., Jacobasch G., Derendorf H., Veit M. (2001). Pharmacokinetics and bioavailability of quercetin glycosides in humans. J. Clin. Pharmacol..

[216] Hollman P.C., de Vries J.H., van Leeuwen S.D., Mengelers M.J., Katan M.B. (1995). Absorption of dietary quercetin glycosides and quercetin in healthy ileostomy volunteers. Am. J. Clin. Nutr..

[217] Olthof M.R., Hollman P.C., Vree T.B., Katan M.B. (2000). Bioavailabilities of quercetin-3-glucoside and quercetin-4’-glucoside do not differ in humans. J. Nutr..

[218] Cao G., Sofic E., Prior R.L. (1997). Antioxidant and prooxidant behavior of flavonoids: Structure-activity relationships. Free Radic. Biol. Med..

[219] Garcia-Lafuente A., Guillamon E., Villares A., Rostagno M.A., Martinez J.A. (2009). Flavonoids as anti-inflammatory agents: Implications in cancer and cardiovascular disease. Inflamm. Res..

[220] Chahar M.K., Sharma N., Dobhal M.P., Joshi Y.C. (2011). Flavonoids: A versatile source of anticancer drugs. Pharmacogn. Rev..

[221] Chang Y.C., Nair M.G. (1995). Metabolism of daidzein and genistein by intestinal bacteria. J. Nat. Prod..

[222] Manach C., Morand C., Crespy V., Demigne C., Texier O., Regerat F., Remesy C. (1998). Quercetin is recovered in human plasma as conjugated derivatives which retain antioxidant properties. FEBS Lett..

[223] Bell J.R., Donovan J.L., Wong R., Waterhouse A.L., German J.B., Walzem R.L., Kasim-Karakas S.E. (2000). (+)-catechin in human plasma after ingestion of a single serving of reconstituted red wine. Am. J. Clin. Nutr..

[224] Hollman P.C.H. (2004). Absorption, bioavailability, and metabolism of flavonoids. Arch. Physiol. Biochem..

[225] Manach C., Williamson G., Morand C., Scalbert A., Remesy C. (2005). Bioavailability and bioefficacy of polyphenols in humans. I. Review of 97 bioavailability studies. Am. J. Clin. Nutr..

[226] Paganga G., Rice-Evans C.A. (1997). The identification of flavonoids as glycosides in human plasma. FEBS Lett..

[227] Cao J., Zhang Y., Chen W., Zhao X. (2010). The relationship between fasting plasma concentrations of selected flavonoids and their ordinary dietary intake. Br. J. Nutr..

[228] Young J.F., Nielsen S.E., Haraldsdottir J., Daneshvar B., Lauridsen S.T., Knuthsen P., Crozier A., Sandstrom B., Dragsted L.O. (1999). Effect of fruit juice intake on urinary quercetin excretion and biomarkers of antioxidative status. Am. J. Clin. Nutr..

[229] Rechner A.R., Kuhnle G., Bremner P., Hubbard G.P., Moore K.P., Rice-Evans C.A. (2002). The metabolic fate of dietary polyphenols in humans. Free Radic. Biol. Med..

[230] Erlund I., Kosonen T., Alfthan G., Maenpaa J., Perttunen K., Kenraali J., Parantainen J., Aro A. (2000). Pharmacokinetics of quercetin from quercetin aglycone and rutin in healthy volunteers. Eur. J. Clin. Pharmacol..

[231] Erlund I., Freese R., Marniemi J., Hakala P., Alfthan G. (2006). Bioavailability of quercetin from berries and the diet. Nutr. Cancer.

[232] Zhang Y., Song T.T., Cunnick J.E., Murphy P.A., Hendrich S. (1999). Daidzein and genistein glucuronides in vitro are weakly estrogenic and activate human natural killer cells at nutritionally relevant concentrations. J. Nutr..

[233] Selma M.V., Espin J.C., Tomas-Barberan F.A. (2009). Interaction between phenolics and gut microbiota: Role in human health. J. Agric. Food Chem..

[234] Sampson L., Rimm E., Hollman P.C., de Vries J.H., Katan M.B. (2002). Flavonol and flavone intakes in us health professionals. J. Am. Diet. Assoc..

[235] Galati G., O’Brien P.J. (2004). Potential toxicity of flavonoids and other dietary phenolics: Significance for their chemopreventive and anticancer properties. Free Radic. Biol. Med..

[236] Pamukcu A.M., Yalciner S., Hatcher J.F., Bryan G.T. (1980). Quercetin, a rat intestinal and bladder carcinogen present in bracken fern (pteridium aquilinum). Cancer Res..

[237] Sahu S.C., Gray G.C. (1993). Interactions of flavonoids, trace metals, and oxygen: Nuclear DNA damage and lipid peroxidation induced by myricetin. Cancer Lett..

[238] Sahu S.C., Gray G.C. (1994). Kaempferol-induced nuclear DNA damage and lipid peroxidation. Cancer Lett..

[239] Dickinson A., Boyon N., Shao A. (2009). Physicians and nurses use and recommend dietary supplements: Report of a survey. Nutr. J..

[240] Brantsaeter A.L., Haugen M., Hagve T.A., Aksnes L., Rasmussen S.E., Julshamn K., Alexander J., Meltzer H.M. (2007). Self-reported dietary supplement use is confirmed by biological markers in the norwegian mother and child cohort study (moba). Ann. Nutr. Metab..

[241] Halliwell B. (2007). Dietary polyphenols: Good, bad, or indifferent for your health?. Cardiovasc. Res..

[242] Popp R., Schimmer O. (1991). Induction of sister-chromatid exchanges (SCE), polyploidy, and micronuclei by plant flavonoids in human lymphocyte cultures. A comparative study of 19 flavonoids. Mutat. Res..

[243] Tiwari A.K., Rao J.M. (2002). Diabetes mellitus and multiple therapeutic approaches of phytochemicals: Present status and future prospects. Curr. Sci. India.

[244] Kaneto H., Katakami N., Matsuhisa M., Matsuoka T.A. (2010). Role of reactive oxygen species in the progression of type 2 diabetes and atherosclerosis. Mediat. Inflamm..

[245] Rolin B., Larsen M.O., Gotfredsen C.F., Deacon C.F., Carr R.D., Wilken M., Knudsen L.B. (2002). The long-acting glp-1 derivative nn2211 ameliorates glycemia and increases beta-cell mass in diabetic mice. Am. J. Physiol. Endocrinol. Metab..

[246] Kotchen T.A. (1996). Attenuation of hypertension by insulin-sensitizing agents. Hypertension.

[247] Derosa G., D’Angelo A., Ragonesi P.D., Ciccarelli L., Piccinni M.N., Pricolo F., Salvadeo S.A., Montagna L., Gravina A., Ferrari I. (2006). Metformin-pioglitazone and metformin-rosiglitazone effects on non-conventional cardiovascular risk factors plasma level in type 2 diabetic patients with metabolic syndrome. J. Clin. Pharm. Ther..

[248] White M.F. (2003). Insulin signaling in health and disease. Science.

[249] Houstis N., Rosen E.D., Lander E.S. (2006). Reactive oxygen species have a causal role in multiple forms of insulin resistance. Nature.

[250] Evans J.L., Goldfine I.D., Maddux B.A., Grodsky G.M. (2002). Oxidative stress and stress-activated signaling pathways: A unifying hypothesis of type 2 diabetes. Endocr. Rev..

[251] Kravchenko L.V., Morozov S.V., Tutel’yan V.A. (2003). Effects of flavonoids on the resistance of microsomes to lipid peroxidation in vitro and ex vivo. Bull. Exp. Biol. Med..

[252] Valenzuela A., Lagos C., Schmidt K., Videla L.A. (1985). Silymarin protection against hepatic lipid-peroxidation induced by acute ethanol intoxication in the rat. Biochem. Pharmacol..

[253] Yao X., Zhu L., Chen Y., Tian J., Wang Y. (2013). In vivo and in vitro antioxidant activity and alpha-glucosidase, alpha-amylase inhibitory effects of flavonoids from cichorium glandulosum seeds. Food Chem..

[254] Yi L., Chen C.Y., Jin X., Zhang T., Zhou Y., Zhang Q.Y., Zhu J.D., Mi M.T. (2012). Differential suppression of intracellular reactive oxygen species-mediated signaling pathway in vascular endothelial cells by several subclasses of flavonoids. Biochimie.

[255] Lin C.M., Chen C.T., Lee H.H., Lin J.K. (2002). Prevention of cellular ros damage by isovitexin and related flavonoids. Planta Medica.

[256] Lotito S.B., Frei B. (2006). Consumption of flavonoid-rich foods and increased plasma antioxidant capacity in humans: Cause, consequence, or epiphenomenon?. Free Radic. Biol. Med..

[257] Brunetti C., Di Ferdinando M., Fini A., Pollastri S., Tattini M. (2013). Flavonoids as antioxidants and developmental regulators: Relative significance in plants and humans. Int. J. Mol. Sci..

[258] Papas A.M. (1996). Determinants of antioxidant status in humans. Lipids.

[259] Gray G.M. (1975). Carbohydrate digestion and absorption. Role of the small intestine. N. Engl. J. Med..

[260] Luna B., Feinglos M.N. (2001). Oral agents in the management of type 2 diabetes mellitus. Am. Fam. Phys..

[261] Cheng A.Y., Fantus I.G. (2005). Oral antihyperglycemic therapy for type 2 diabetes mellitus. Can. Med. Assoc. J..

[262] Li Y.Q., Zhou F.C., Gao F., Bian J.S., Shan F. (2009). Comparative evaluation of quercetin, isoquercetin and rutin as inhibitors of alpha-glucosidase. J. Agric. Food. Chem..

[263] Zhang X., Liu Z., Bi X., Liu J., Li W., Zhao Y. (2013). Flavonoids and its derivatives from callistephus chinensis flowers and their inhibitory activities against alpha-glucosidase. EXCLI J..

[264] Pereira D.F., Cazarolli L.H., Lavado C., Mengatto V., Figueiredo M.S.R.B., Guedes A., Pizzolatti M.G., Silva F.R.M.B. (2011). Effects of flavonoids on alpha-glucosidase activity: Potential targets for glucose homeostasis. Nutrition.

[265] Cermak R., Landgraf S., Wolffram S. (2004). Quercetin glucosides inhibit glucose uptake into brush-border-membrane vesicles of porcine jejunum. Br. J. Nutr..

[266] Song J., Kwon O., Chen S.L., Daruwala R., Eck P., Park J.B., Levine M. (2002). Flavonoid inhibition of sodium-dependent vitamin c transporter 1 (SVCT1) and glucose transporter isoform 2 (GLUT2), intestinal transporters for vitamin c and glucose. J. Biol. Chem..

[267] Goto T., Horita M., Nagai H., Nagatomo A., Nishida N., Matsuura Y., Nagaoka S. (2012). Tiliroside, a glycosidic flavonoid, inhibits carbohydrate digestion and glucose absorption in the gastrointestinal tract. Mol. Nutr. Food Res..

[268] de la Garza A.L., Etxeberria U., Lostao M.P., San Roman B., Barrenetxe J., Martinez J.A., Milagro F.I. (2013). Helichrysum and grapefruit extracts inhibit carbohydrate digestion and absorption, improving postprandial glucose levels and hyperinsulinemia in rats. J. Agric. Food Chem..

[269] Kim J.S., Kwon C.S., Son K.H. (2000). Inhibition of alpha-glucosidase and amylase by luteolin, a flavonoid. Biosci. Biotechnol. Biochem..

[270] Matsui T., Kobayashi M., Hayashida S., Matsumoto K. (2002). Luteolin, a flavone, does not suppress postprandial glucose absorption through an inhibition of alpha-glucosidase action. Biosci. Biotechnol. Biochem..

[271] Kobayashi Y., Suzuki M., Satsu H., Arai S., Hara Y., Suzuki K., Miyamoto Y., Shimizu M. (2000). Green tea polyphenols inhibit the sodium-dependent glucose transporter of intestinal epithelial cells by a competitive mechanism. J. Agric. Food Chem..

[272] Shimizu M., Kobayashi Y., Suzuki M., Satsu H., Miyamoto Y. (2000). Regulation of intestinal glucose transport by tea catechins. Biofactors.

[273] Johnston K.L., Clifford M.N., Morgan L.M. (2002). Possible role for apple juice phenolic, compounds in the acute modification of glucose tolerance and gastrointestinal hormone secretion in humans. J. Sci. Food Agric..

[274] Holt S., Jong V.D., Faramus E., Lang T., Brand Miller J. (2003). A bioflavonoid in sugar cane can reduce the postprandial glycaemic response to a high-gi starchy food. Asia Pac. J. Clin. Nutr..

[275] Grussu D., Stewart D., McDougall G.J. (2011). Berry polyphenols inhibit alpha-amylase in vitro: Identifying active components in rowanberry and raspberry. J. Agric. Food Chem..

[276] Alzaid F., Cheung H.M., Preedy V.R., Sharp P.A. (2013). Regulation of glucose transporter expression in human intestinal caco-2 cells following exposure to an anthocyanin-rich berry extract. PLoS ONE.

[277] Zhang A.J., Rimando A.M., Fish W., Mentreddy S.R., Mathews S.T. (2012). Serviceberry [amelanchier alnifolia (nutt.) nutt. Ex. M. Roem (rosaceae)] leaf extract inhibits mammalian alpha-glucosidase activity and suppresses postprandial glycemic response in a mouse model of diet-induced obesity and hyperglycemia. J. Ethnopharmacol..

[278] Torronen R., Sarkkinen E., Tapola N., Hautaniemi E., Kilpi K., Niskanen L. (2010). Berries modify the postprandial plasma glucose response to sucrose in healthy subjects. Br. J. Nutr..

[279] Torronen R., McDougall G.J., Dobson G., Stewart D., Hellstrom J., Mattila P., Pihlava J.M., Koskela A., Karjalainen R. (2012). Fortification of blackcurrant juice with crowberry: Impact on polyphenol composition, urinary phenolic metabolites, and postprandial glycemic response in healthy subjects. J. Funct. Foods.

[280] Clegg M.E., Pratt M., Meade C.M., Henry C.J.K. (2011). The addition of raspberries and blueberries to a starch-based food does not alter the glycaemic response. Br. J. Nutr..

[281] Wilson T., Singh A.P., Vorsa N., Goettl C.D., Kittleson K.M., Roe C.M., Kastello G.M., Ragsdale F.R. (2008). Human glycemic response and phenolic content of unsweetened cranberry juice. J. Med. Food.

[282] Wilson T., Luebke J.L., Morcomb E.F., Carrell E.J., Leveranz M.C., Kobs L., Schmidt T.P., Limburg P.J., Vorsa N., Singh A.P. (2010). Glycemic responses to sweetened dried and raw cranberries in humans with type 2 diabetes. J. Food Sci..

[283] Lo Piparo E., Scheib H., Frei N., Williamson G., Grigorov M., Chou C.J. (2008). Flavonoids for controlling starch digestion: Structural requirements for inhibiting human alpha-amylase. J. Med. Chem..

[284] Yang J.P., He H., Lu Y.H. (2014). Four flavonoid compounds from phyllostachys edulis leaf extract retard the digestion of starch and its working mechanisms. J. Agric. Food Chem..

[285] Zhao F.Q., Keating A.F. (2007). Functional properties and genomics of glucose transporters. Curr. Genom..

[286] Uldry M., Thorens B. (2004). The SLC2 family of facilitated hexose and polyol transporters. Pflug. Arch. Eur. J. Physiol..

[287] Zaid H., Antonescu C.N., Randhawa V.K., Klip A. (2008). Insulin action on glucose transporters through molecular switches, tracks and tethers. Biochem. J..

[288] Dugani C.B., Randhawa V.K., Cheng A.W.P., Patel N., Klip A. (2008). Selective regulation of the perinuclear distribution of glucose transporter 4 (GLUT4) by insulin signals in muscle cells. Eur. J. Cell Biol..

[289] Liu W., Hsin C., Tang F. (2009). A molecular mathematical model of glucose mobilization and uptake. Math. Biosci..

[290] Yamaguchi S., Katahira H., Ozawa S., Nakamichi Y., Tanaka T., Shimoyama T., Takahashi K., Yoshimoto K., Imaizumi M.O., Nagamatsu S. (2005). Activators of AMP-activated protein kinase enhance GLUT4 translocation and its glucose transport activity in 3T3-L1 adipocytes. Am. J. Physiol. Endocrinol. Metab..

[291] Musi N., Hayashi T., Fujii N., Hirshman M.F., Witters L.A., Goodyear L.J. (2001). Amp-activated protein kinase activity and glucose uptake in rat skeletal muscle. Am. J. Physiol. Endocrinol. Metab..

[292] Pinent M., Blay M., Blade M.C., Salvado M.J., Arola L., Ardevol A. (2004). Grape seed-derived procyanidins have an antihyperglycemic effect in streptozotocin-induced diabetic rats and insulinomimetic activity in insulin-sensitive cell lines. Endocrinology.

[293] Montagut G., Onnockx S., Vaque M., Blade C., Blay M., Fernandez-Larrea J., Pujadas G., Salvado M.J., Arola L., Pirson I. (2010). Oligomers of grape-seed procyanidin extract activate the insulin receptor and key targets of the insulin signaling pathway differently from insulin. J. Nutr. Biochem..

[294] Montagut G., Blade C., Blay M., Fernandez-Larrea J., Pujadas G., Salvado M.J., Arola L., Pinent M., Ardevol A. (2010). Effects of a grapeseed procyanidin extract (GSPE) on insulin resistance. J. Nutr. Biochem..

[295] Cao H., Hininger-Favier I., Kelly M.A., Benaraba R., Dawson H.D., Coves S., Roussel A.M., Anderson R.A. (2007). Green tea polyphenol extract regulates the expression of genes involved in glucose uptake and insulin signaling in rats fed a high fructose diet. J. Agric. Food Chem..

[296] Zhang Z.F., Li Q., Liang J., Dai X.Q., Ding Y., Wang J.B., Li Y. (2010). Epigallocatechin-3-O-gallate (EGCG) protects the insulin sensitivity in rat l6 muscle cells exposed to dexamethasone condition. Phytomed. Int. J. Phytother. Phytopharmacol..

[297] Lee M.S., Kim C.H., Hoang D.M., Kim H.Y., Sohn C.B., Kim M.R., Ahn J.S. (2009). Genistein-derivatives from tetracera scandens stimulate glucose-uptake in l6 myotubes. Biol. Pharm. Bull..

[298] Zhang W.Y., Lee J.J., Kim Y., Kim I.S., Han J.H., Lee S.G., Ahn M.J., Jung S.H., Myung C.S. (2012). Effect of eriodictyol on glucose uptake and insulin resistance in vitro. J. Agric. Food. Chem..

[299] Zhang W.Y., Lee J.J., Kim I.S., Kim Y., Park J.S., Myung C.S. (2010). 7-O-methylaromadendrin stimulates glucose uptake and improves insulin resistance in vitro. Biol. Pharm. Bull..

[300] Fang X.K., Gao J., Zhu D.N. (2008). Kaempferol and quercetin isolated from euonymus alatus improve glucose uptake of 3T3-L1 cells without adipogenesis activity. Life Sci..

[301] Miyata Y., Tanaka H., Shimada A., Sato T., Ito A., Yamanouchi T., Kosano H. (2011). Regulation of adipocytokine secretion and adipocyte hypertrophy by polymethoxyflavonoids, nobiletin and tangeretin. Life Sci..

[302] Sharma A.K., Bharti S., Ojha S., Bhatia J., Kumar N., Ray R., Kumari S., Arya D.S. (2011). Up-regulation of ppar gamma, heat shock protein-27 and -72 by naringin attenuates insulin resistance, beta-cell dysfunction, hepatic steatosis and kidney damage in a rat model of type 2 diabetes. Br. J. Nutr..

[303] Lv X.W., Li J., Jin Y., Zhang L., Wang J.Q. (2009). Effects and mechanisms of total flavonoids of litsea coreana on insulin resistance in rats with hyperlipidemia. J. Chin. Med. Mater..

[304] Lu Y.X., Zhang Q., Li J., Sun Y.X., Wang L.Y., Cheng W.M., Hu X.Y. (2010). Antidiabetic effects of total flavonoids from litsea coreana leve on fat-fed, streptozotocin-induced type 2 diabetic rats. Am. J. Chin. Med..

[305] Goto T., Teraminami A., Lee J.Y., Ohyama K., Funakoshi K., Kim Y.I., Hirai S., Uemura T., Yu R., Takahashi N. (2012). Tiliroside, a glycosidic flavonoid, ameliorates obesity-induced metabolic disorders via activation of adiponectin signaling followed by enhancement of fatty acid oxidation in liver and skeletal muscle in obese-diabetic mice. J. Nutr. Biochem..

[306] Stoffers D.A. (2004). The development of beta-cell mass: Recent progress and potential role of glp-1. Horm. Metab. Res..

[307] Tourrel C., Bailbe D., Lacorne M., Meile M.J., Kergoat M., Portha B. (2002). Persistent improvement of type 2 diabetes in the goto-kakizaki rat model by expansion of the beta-cell mass during the prediabetic period with glucagon-like peptide-1 or exendin-4. Diabetes.

[308] Sakuraba H., Mizukami H., Yagihashi N., Wada R., Hanyu C., Yagihashi S. (2002). Reduced beta-cell mass and expression of oxidative stress-related DNA damage in the islet of japanese type ii diabetic patients. Diabetologia.

[309] Marchetti P., Del Guerra S., Marselli L., Lupi R., Masini M., Pollera M., Bugliani M., Boggi U., Vistoli F., Mosca F. (2004). Pancreatic islets from type 2 diabetic patients have functional defects and increased apoptosis that are ameliorated by metformin. J. Clin. Endocrinol. Metab..

[310] Cozar-Castellano I., Fiaschi-Taesch N., Bigatel T.A., Takane K.K., Garcia-Ocana A., Vasavada R., Stewart A.F. (2006). Molecular control of cell cycle progression in the pancreatic beta-cell. Endocr. Rev..

[311] Butler A.E., Janson J., Bonner-Weir S., Ritzel R., Rizza R.A., Butler P.C. (2003). Beta-cell deficit and increased beta-cell apoptosis in humans with type 2 diabetes. Diabetes.

[312] Chang-Chen K.J., Mullur R., Bernal-Mizrachi E. (2008). Beta-cell failure as a complication of diabetes. Rev. Endocr. Metab. Dis..

[313] Lenzen S. (2008). The mechanisms of alloxan- and streptozotocin-induced diabetes. Diabetologia.

[314] Vessal M., Hemmati M., Vasei M. (2003). Antidiabetic effects of quercetin in streptozocin-induced diabetic rats. Comp. Biochem. Physiol. Toxicol. Pharmacol..

[315] Coskun O., Kanter M., Korkmaz A., Oter S. (2005). Quercetin, a flavonoid antioxidant, prevents and protects streptozotocin-induced oxidative stress and beta-cell damage in rat pancreas. Pharmacol. Res..

[316] Choi J.S., Yokozawa T., Oura H. (1991). Improvement of hyperglycemia and hyperlipemia in streptozotocin-diabetic rats by a methanolic extract of prunus-davidiana stems and its main component prunin. Planta Medica.

[317] Chakravarthy B.K., Gupta S., Gode K.D. (1982). Functional beta cell regeneration in the islets of pancreas in alloxan induced diabetic rats by (-)-epicatechin. Life Sci..

[318] Martin M.A., Fernandez-Millan E., Ramos S., Bravo L., Goya L. (2014). Cocoa flavonoid epicatechin protects pancreatic beta cell viability and function against oxidative stress. Mol. Nutr. Food Res..

[319] Esmaeili M.A., Zohari F., Sadeghi H. (2009). Antioxidant and protective effects of major flavonoids from teucrium polium on beta-cell destruction in a model of streptozotocin-induced diabetes. Planta Medica.

[320] Kwon D.Y., Daily J.W., Kim H.J., Park S. (2010). Antidiabetic effects of fermented soybean products on type 2 diabetes. Nutr. Res..

[321] Hanhineva K., Torronen R., Bondia-Pons I., Pekkinen J., Kolehmainen M., Mykkanen H., Poutanen K. (2010). Impact of dietary polyphenols on carbohydrate metabolism. Int. J. Mol. Sci..

[322] Choi M.S., Jung U.J., Yeo J., Kim M.J., Lee M.K. (2008). Genistein and daidzein prevent diabetes onset by elevating insulin level and altering hepatic gluconeogenic and lipogenic enzyme activities in non-obese diabetic (NOD) mice. Diabetes/Metab. Res. Rev..

[323] Guo T.L., Germolec D.R., Zheng J.F., Kooistra L., Auttachoat W., Smith M.J., White K.L., Elmore S.A. (2015). Genistein protects female nonobese diabetic mice from developing type 1 diabetes when fed a soy- and alfalfa-free diet. Toxicol. Pathol..

[324] Fu Z., Zhang W., Zhen W., Lum H., Nadler J., Bassaganya-Riera J., Jia Z., Wang Y., Misra H., Liu D. (2010). Genistein induces pancreatic beta-cell proliferation through activation of multiple signaling pathways and prevents insulin-deficient diabetes in mice. Endocrinology.

[325] Fu Z., Gilbert E.R., Pfeiffer L., Zhang Y., Fu Y., Liu D. (2012). Genistein ameliorates hyperglycemia in a mouse model of nongenetic type 2 diabetes. Appl. Physiol. Nutr. Metab..

[326] Liu D., Zhen W., Yang Z., Carter J.D., Si H., Reynolds K.A. (2006). Genistein acutely stimulates insulin secretion in pancreatic beta-cells through a camp-dependent protein kinase pathway. Diabetes.

[327] Gilbert E.R., Liu D. (2013). Anti-diabetic functions of soy isoflavone genistein: Mechanisms underlying its effects on pancreatic beta-cell function. Food Funct..

[328] Liu Y.J., Zhan J., Liu X.L., Wang Y., Ji J., He Q.Q. (2014). Dietary flavonoids intake and risk of type 2 diabetes: A meta-analysis of prospective cohort studies. Clin. Nutr..

[329] Nettleton J.A., Harnack L.J., Scrafford C.G., Mink P.J., Barraj L.M., Jacobs D.R. (2006). Dietary flavonoids and flavonoid-rich foods are not associated with risk of type 2 diabetes in postmenopausal women. J. Nutr..

[330] Zamora-Ros R., Forouhi N.G., Sharp S.J., Gonzalez C.A., Buijsse B., Guevara M., van der Schouw Y.T., Amiano P., Boeing H., Bredsdorff L. (2014). Dietary intakes of individual flavanols and flavonols are inversely associated with incident type 2 diabetes in european populations. J. Nutr..

[331] Knekt P., Kumpulainen J., Jarvinen R., Rissanen H., Heliovaara M., Reunanen A., Hakulinen T., Aromaa A. (2002). Flavonoid intake and risk of chronic diseases. Am. J. Clin. Nutr..

[332] Huxley R., Lee C.M., Barzi F., Timmermeister L., Czernichow S., Perkovic V., Grobbee D.E., Batty D., Woodward M. (2009). Coffee, decaffeinated coffee, and tea consumption in relation to incident type 2 diabetes mellitus: A systematic review with meta-analysis. Arch. Intern. Med..

[333] Yang W.S., Wang W.Y., Fan W.Y., Deng Q., Wang X. (2014). Tea consumption and risk of type 2 diabetes: A dose-response meta-analysis of cohort studies. Br. J. Nutr..

[334] Iso H., Date C., Wakai K., Fukui M., Tamakoshi A., Grp J.S. (2006). The relationship between green tea and total caffeine intake and risk for self-reported type 2 diabetes among japanese adults. Ann. Intern. Med..

[335] InterAct C., van Woudenbergh G.J., Kuijsten A., Drogan D., van der A.D., Romaguera D., Ardanaz E., Amiano P., Barricarte A., Beulens J.W. (2012). Tea consumption and incidence of type 2 diabetes in europe: The epic-interact case-cohort study. PLoS ONE.

[336] Jing Y., Han G., Hu Y., Bi Y., Li L., Zhu D. (2009). Tea consumption and risk of type 2 diabetes: A meta-analysis of cohort studies. J. Gen. Intern. Med..

[337] Panagiotakos D.B., Lionis C., Zeimbekis A., Gelastopoulou K., Papairakleous N., Das U.N., Polychronopoulos E. (2009). Long-term tea intake is associated with reduced prevalence of (type 2) diabetes mellitus among elderly people from mediterranean islands: Medis epidemiological study. Yonsei Med. J..

[338] Muraki I., Imamura F., Manson J.E., Hu F.B., Willett W.C., van Dam R.M., Sun Q. (2013). Fruit consumption and risk of type 2 diabetes: Results from three prospective longitudinal cohort studies. BMJ.

[339] Wedick N.M., Pan A., Cassidy A., Rimm E.B., Sampson L., Rosner B., Willett W., Hu F.B., Sun Q., van Dam R.M. (2012). Dietary flavonoid intakes and risk of type 2 diabetes in us men and women. Am. J. Clin. Nutr..

[340] Pandey K.B., Rizvi S.I. (2009). Plant polyphenols as dietary antioxidants in human health and disease. Oxid. Med. Cell. Longev..

[341] Fu Z., Zhen W., Yuskavage J., Liu D. (2010). Epigallocatechin gallate delays the onset of type 1 diabetes in spontaneous non-obese diabetic mice. Br. J. Nutr..

[342] Seeram N.P., Adams L.S., Hardy M.L., Heber D. (2004). Total cranberry extract versus its phytochemical constituents: Antiproliferative and synergistic effects against human tumor cell lines. J. Agric. Food Chem..

[343] Yang J., Liu R.H. (2009). Synergistic effect of apple extracts and quercetin 3-beta-d-glucoside combination on antiproliferative activity in mcf-7 human breast cancer cells in vitro. J. Agric. Food Chem..

[344] Huseini H.F., Larijani B., Heshmat R., Fakhrzadeh H., Radjabipour B., Toliat T., Raza M. (2006). The efficacy of silybum marianum (L.) gaertn. (silymarin) in the treatment of type ii diabetes: A randomized, double-blind, placebo-controlled, clinical trial. Phytother. Res..

[345] Lirussi F., Beccarello A., Zanette G., De Monte A., Donadon V., Velussi M., Crepaldi G. (2002). Silybin-beta-cyclodextrin in the treatment of patients with diabetes mellitus and alcoholic liver disease. Efficacy study of a new preparation of an anti-oxidant agent. Diabetes Nutr. Metab..

[346] Chambers B.K., Camire M.E. (2003). Can cranberry supplementation benefit adults with type 2 diabetes?. Diabetes Care.

[347] Simeonov S.B., Botushanov N.P., Karahanian E.B., Pavlova M.B., Husianitis H.K., Troev D.M. (2002). Effects of aronia melanocarpa juice as part of the dietary regimen in patients with diabetes mellitus. Folia Medica.

[348] Kar P., Laight D., Rooprai H.K., Shaw K.M., Cummings M. (2009). Effects of grape seed extract in type 2 diabetic subjects at high cardiovascular risk: A double blind randomized placebo controlled trial examining metabolic markers, vascular tone, inflammation, oxidative stress and insulin sensitivity. Diabet. Med..

[349] Zunino S.J., Peerson J.M., Freytag T.L., Breksa A.P., Bonnel E.L., Woodhouse L.R., Storms D.H. (2014). Dietary grape powder increases IL-1beta and IL-6 production by lipopolysaccharide-activated monocytes and reduces plasma concentrations of large ldl and large ldl-cholesterol particles in obese humans. Br. J. Nutr..

[350] Qin Y., Xia M., Ma J., Hao Y., Liu J., Mou H., Cao L., Ling W. (2009). Anthocyanin supplementation improves serum LDL- and HDL-cholesterol concentrations associated with the inhibition of cholesteryl ester transfer protein in dyslipidemic subjects. Am. J. Clin. Nutr..

[351] Zhu Y., Ling W., Guo H., Song F., Ye Q., Zou T., Li D., Zhang Y., Li G., Xiao Y. (2013). Anti-inflammatory effect of purified dietary anthocyanin in adults with hypercholesterolemia: A randomized controlled trial. Nutr. Metab. Cardiovasc. Dis..

[352] Fukino Y., Shimbo M., Aoki N., Okubo T., Iso H. (2005). Randomized controlled trial for an effect of green tea consumption on insulin resistance and inflammation markers. J. Nutr. Sci. Vitaminol..

[353] Ryu O.H., Lee J., Lee K.W., Kim H.Y., Seo J.A., Kim S.G., Kim N.H., Baik S.H., Choi D.S., Choi K.M. (2006). Effects of green tea consumption on inflammation, insulin resistance and pulse wave velocity in type 2 diabetes patients. Diabetes Res. Clin. Pract..

[354] Mackenzie T., Leary L., Brooks W.B. (2007). The effect of an extract of green and black tea on glucose control in adults with type 2 diabetes mellitus: Double-blind randomized study. Metab. Clin. Exp..

[355] Hsu C.H., Liao Y.L., Lin S.C., Tsai T.H., Huang C.J., Chou P. (2011). Does supplementation with green tea extract improve insulin resistance in obese type 2 diabetics? A randomized, double-blind, and placebo-controlled clinical trial. Altern. Med. Rev..

[356] Nagao T., Meguro S., Hase T., Otsuka K., Komikado M., Tokimitsu I., Yamamoto T., Yamamoto K. (2009). A catechin-rich beverage improves obesity and blood glucose control in patients with type 2 diabetes. Obesity.

[357] Takahashi M., Miyashita M., Suzuki K., Bae S.R., Kim H.K., Wakisaka T., Matsui Y., Takeshita M., Yasunaga K. (2014). Acute ingestion of catechin-rich green tea improves postprandial glucose status and increases serum thioredoxin concentrations in postmenopausal women. Br. J. Nutr..

[358] Larsen N., Vogensen F.K., van den Berg F.W., Nielsen D.S., Andreasen A.S., Pedersen B.K., Al-Soud W.A., Sørensen S.J., Hansen L.H., Jakobsen M. (2010). Gut microbiota in human adults with type 2 diabetes differs from non-diabetic adults. PLoS ONE.

[359] Zhang X., Shen D., Fang Z., Jie Z., Qiu X., Zhang C., Chen Y., Ji L. (2013). Human gut microbiota changes reveal the progression of glucose intolerance. PLoS ONE.

[360] Qin J., Li Y., Cai Z., Li S., Zhu J., Zhang F., Liang S., Zhang W., Guan Y., Shen D. (2012). A metagenome-wide association study of gut microbiota in type 2 diabetes. Nature.

[361] Egshatyan L., Kashtanova D., Popenko A., Tkacheva O., Tyakht A., Alexeev D., Karamnova N., Kostryukova E., Babenko V., Vakhitova M. (2016). Gut microbiota and diet in patients with different glucose tolerance. Endocr. Connect..

[362] Cani P.D., Possemiers S., Van de Wiele T., Guiot Y., Everard A., Rottier O., Geurts L., Naslain D., Neyrinck A., Lambert D.M. (2009). Changes in gut microbiota control inflammation in obese mice through a mechanism involving GLP-2-driven improvement of gut permeability. Gut.

[363] Cani P.D., Neyrinck A., Fava F., Knauf C., Burcelin R., Tuohy K., Gibson G., Delzenne N. (2007). Selective increases of bifidobacteria in gut microflora improve high-fat-diet-induced diabetes in mice through a mechanism associated with endotoxaemia. Diabetologia.

[364] Yan J., Zhang G., Pan J., Wang Y. (2014). α-glucosidase inhibition by luteolin: Kinetics, interaction and molecular docking. Int. J. Biol. Macromol..

[365] Braune A., Blaut M. (2016). Bacterial species involved in the conversion of dietary flavonoids in the human gut. Gut Microb..

